# A reactor for *in situ*, time-resolved neutron diffraction studies of microwave-induced rapid solid-state chemical reactions

**DOI:** 10.1098/rsta.2024.0065

**Published:** 2025-05-22

**Authors:** Ross George Bell McFadzean, Ronald Smith, Timothy David Drysdale, Duncan H Gregory

**Affiliations:** ^1^ Chemistry, University of Glasgow, Glasgow, UK; ^2^ ISIS Pulsed Neutron and Muon Source, Didcot, Oxfordshire, UK; ^3^ Institute for Digital Communications, University of Edinburgh School of Engineering, Edinburgh, UK

**Keywords:** microwaves, solid state, materials, synthesis, chalcogenides, neutrons

## Abstract

Utilizing direct microwave- (MW)-induced heating in solid-state synthesis yields the clear benefits of greatly reduced reaction times and lower energy requirements as compared with conventional methods. Here, we describe a bespoke single-mode cavity (SMC) MW reactor designed to operate within a neutron beamline that allowed powder diffraction data to be collected from materials *in situ* as they were heated using MWs. The unique set-up was used to investigate the rapid solid-state synthesis of the binary metal chalcogenide thermoelectric (TE) materials Bi_2_Se_3_, Bi_2_Te_3_, Sb_2_Se_3_ and Sb_2_Te_3_. The resultant time-resolved diffraction data from each synthesis were time-sliced post-reaction into segments covering periods of tens of seconds, enabling the reaction progression to be visualized as colourmap plots. This technique enabled the accurate tracking of polycrystalline structure formation and a quantitative analysis of phase fractions during the accelerated heating and subsequent cooling stages of each reaction. Our investigations have also revealed some of the present limitations of rapid *in situ* neutron diffraction techniques and how these might be remedied.

This article is part of the discussion meeting issue ‘Microwave science in sustainability’.

## 1. Introduction

With contemporary global issues of climate change, pollution and associated risks to human health, there is a growing impetus behind low-carbon, energy-efficient industrial processes. A growing group of such energy-efficient approaches is gradually transitioning from lab to plant scale [1–6]. Given the equally important emphasis on renewables and sustainable energy generation, new industrial manufacturing technologies towards ‘green’ production of energy conversion and storage materials, which themselves are low-cost and Earth-abundant, are a priority [7]. Traditional synthesis of materials such as ceramics involves high-temperature processes requiring long durations (hours to days) to complete, and methods that are dependent on fossil fuels to operate.

One viable alternative to such conventional approaches is to adopt alternative procedures driven sustainably by renewably generated electricity. Microwave (MW) heating methods fall within this category and can offer the synthesis of materials at high purity sustainably and economically [8]. Particularly attractive is the prospect of rapid processing (higher throughput for both batch and continuous operations), enhanced energy efficiency and reduced running costs as compared with conventional high-temperature methods. We have investigated the prospects of this at the lab scale for several decades [8–11]. Unlike conventional heating, MW energy (and predominantly the electrical component of the MW field) is selectively absorbed and converted rapidly into heat, enabling instantaneous volumetric heating of materials with the appropriate dielectric or conducting properties [8,12,13]. In fact, the rapid MW heating of a huge selection of powders can be exploited reproducibly in solid-state syntheses either by direct MW heating or by indirect heating using susceptors with the appropriate properties (high-dielectric loss) to couple with the electromagnetic field [8,14–16]. Careful experimental design can also significantly broaden the synthesis possibilities. For example, whereas bulk metals reflect MWs due to their small skin (penetration) depth [12,13], using metal particles at the submicron regime (below the skin depth), results in tremendously increased MW absorption and remarkably high heating rates, as was demonstrated in some of the original pioneering MW materials synthesis experiments [17,18]. The same principles of rapid heating to high temperature can be used for post-synthesis materials processing, which is often required in the sintering of metals or ceramics [19,20]. Moreover, unlike some other emerging energy-efficient synthesis/processing methodologies, through control of rapid heating and cooling, MWs offer access to new (e.g. metastable) materials with unexpected structures and properties coupled with the advantages of engineering morphology from nanostructured particles through bulk powders to single crystals and monoliths.

Despite significant technical advances in the development of MW heating in solid-state synthesis, progress in the area has not matched the larger growth of solution-based or solvothermal reaction systems. This is due partly to the lack of commercial laboratory-scale reactors designed for solid-state synthesis compared with those manufactured specifically for the solution-state synthesis of organic reagents, for example. There are also some fundamental challenges such as the difficulty of accurately monitoring and recording the temperature within solid samples [8]. Moreover, the underlying mechanisms of MW-induced solid-state reactions are largely unknown; there are sizeable gaps in understanding both regarding the reaction pathways followed and the control parameters required to achieve them. Characteristics such as the increased rate of MW- versus conventionally heated reactions are often assigned to ‘non-thermal effects’ related to MW volumetric heating as opposed to conventional heat-transfer mechanisms [21]. The lack of understanding of both interaction and reaction mechanisms is perhaps not surprising considering the sizeable barriers to probing MW-driven synthesis *in situ*. Any characterization technique has to be both penetrating (allowing access to samples within an electromagnetic field and associated containment infrastructure) and capable of rapid time-resolved measurement of distinctive/representative properties/traits of a sample.

For the heating of crystalline solids, powder neutron diffraction (PND) has already proved to be an invaluable tool enabling the determination of structure within complex sample environments, under sometimes challenging physical conditions over variable experimental durations. In actuality, there is a precedent that suggests PND could be the method of choice for MW-heated sample systems. Harrison *et al.* pioneered the use of *in situ* neutron diffraction to study MW versus conventional heating of crystals [22]. The study focused on the structural characterization of a single-crystal sample of aspirin cooled in a closed-cycle refrigerator. In one case, the crystal was subsequently heated conventionally to a higher temperature, while in another, MW heating was applied. Structure refinement showed that at 100 K the MW-heated crystal exhibited thermal displacement parameters similar to those of the same crystal when heating conventionally to 300 K. This fundamental study thus indicated the increased atomic mobility achieved in a MW field; a phenomenon that was not simply temperature dependent.

Motivated by the desire to understand not only how crystalline structures behave when heated in a MW field but also by the need to comprehend how *chemical reactions* themselves are driven by such an environment, we sought to design a MW synthesis reactor that could be used for time-resolved studies of solid-state *in situ* reactions. Inspired by the original studies of Harrison *et al*. [22], we therefore, concentrated on developing a reactor that could be utilized *in situ* on a neutron beamline for diffraction experiments. With the vast improvements in detector and software technology made since the turn of the millennium, the neutron powder diffractometer Polaris at the ISIS Neutron and Muon Source (at the Rutherford Appleton Laboratory, Harwell in the UK) was considered an excellent candidate for installing such a piece of equipment.

Herein, we describe the process of designing, building and installing the first single-mode cavity (SMC) MW reactor for the study of solid-state materials synthesis *in situ* on a neutron beamline. We also describe the initial commissioning experiments that were performed to validate the reactor. For this purpose, and mindful of our ambition to produce sustainable energy materials sustainably, we selected the synthesis of two families of state-of-the-art thermoelectric (TE) materials; bismuth and antimony chalcogenides M_2_X_3_ (M = Bi, Sb; X = Se, Te). Not only do both families represent important commercially valuable materials, but also each can be prepared from starting materials that couple readily with a MW field. Indeed, there is precedent for the successful MW synthesis of such materials, albeit using less sophisticated multimode cavity (MMC) reactors. Sb_2_Te_3_ presents as an intrinsic *p*-type TE due to a high-thermal lattice conductivity, *κ_l_*, combined with *anti*site defects [23]. Both Sb_2_Se_3_ and Sb_2_Te_3_ have been synthesized by direct MW heating in a MMC reactor (which limits control and can make reproducing results difficult) [14]. The highest *zT* values for Sb_2_Te_3_-related materials (*ca* 1 at 400 K) can be achieved by complex alloying processes, including spark plasma sintering [24]. Bi_2_Te_3_ and associated alloys have been among the best-performing TEs for many years and the telluride can be tuned to be either *n*- or *p*-type by adjusting the BiTe ratio [25]; *p*-type Bi_2_Te_3_ is commercially available with typically *zT* = 1.11 at 323−348 K [26]. Both Bi_2_Se_3_ and Bi_2_Te_3_ have been prepared using direct MW heating (for 600 s) in an 800 W domestic MW oven with periodic shaking [27,28]. Bi_2_Se_3_ and Bi_2_Te_3_ were also prepared at 1000 W for 4 × 60 s cycles using an MMC reactor [14]. With these details in mind, our objective was to synthesize the binary metal chalcogenide TE materials through the direct MW-induced heating of the elemental powders. These reactions, we anticipated, would provide an exciting and challenging case study for *in situ* PND experiments using the SMC reactor. By matching the MW power absorption profiles for the samples with their time-resolved diffractograms, it should be possible to identify the key steps in the respective synthesis pathways and, ultimately, determine how and why they occur.

## 2. Experimental

### (a) SMC reactor design

**Figure 1.**
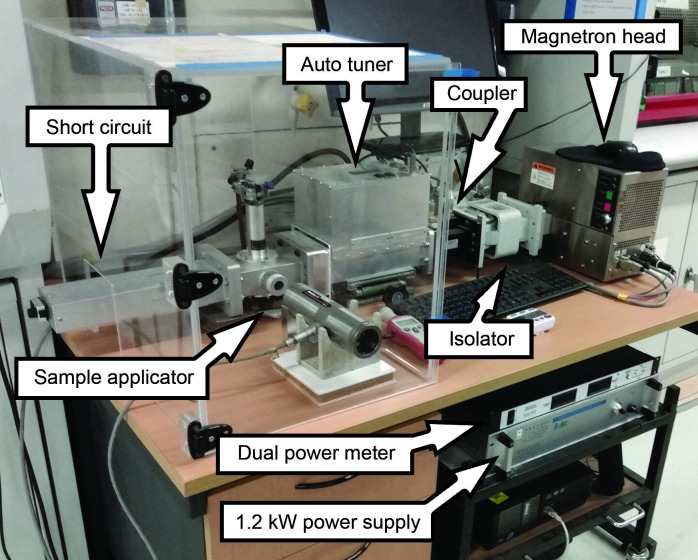
Photograph of the ‘benchtop’ lab-based SMC MW reactor (linear configuration). The respective components are labelled.

Unlike the cavity in a domestic MW oven, which is designed for multiple MW modes, i.e. a MMC reactor, we designed a bespoke SMC reactor built from modular components to produce a single standing wave heating mode within the sample applicator. Initially, a lab-scale, benchtop instrument was constructed for testing purposes; the components were connected in series using sections of aluminium WR340 waveguide (WG) and flanges (figure 1). Further details are provided in the electronic supplementary material.

**Figure 2.**
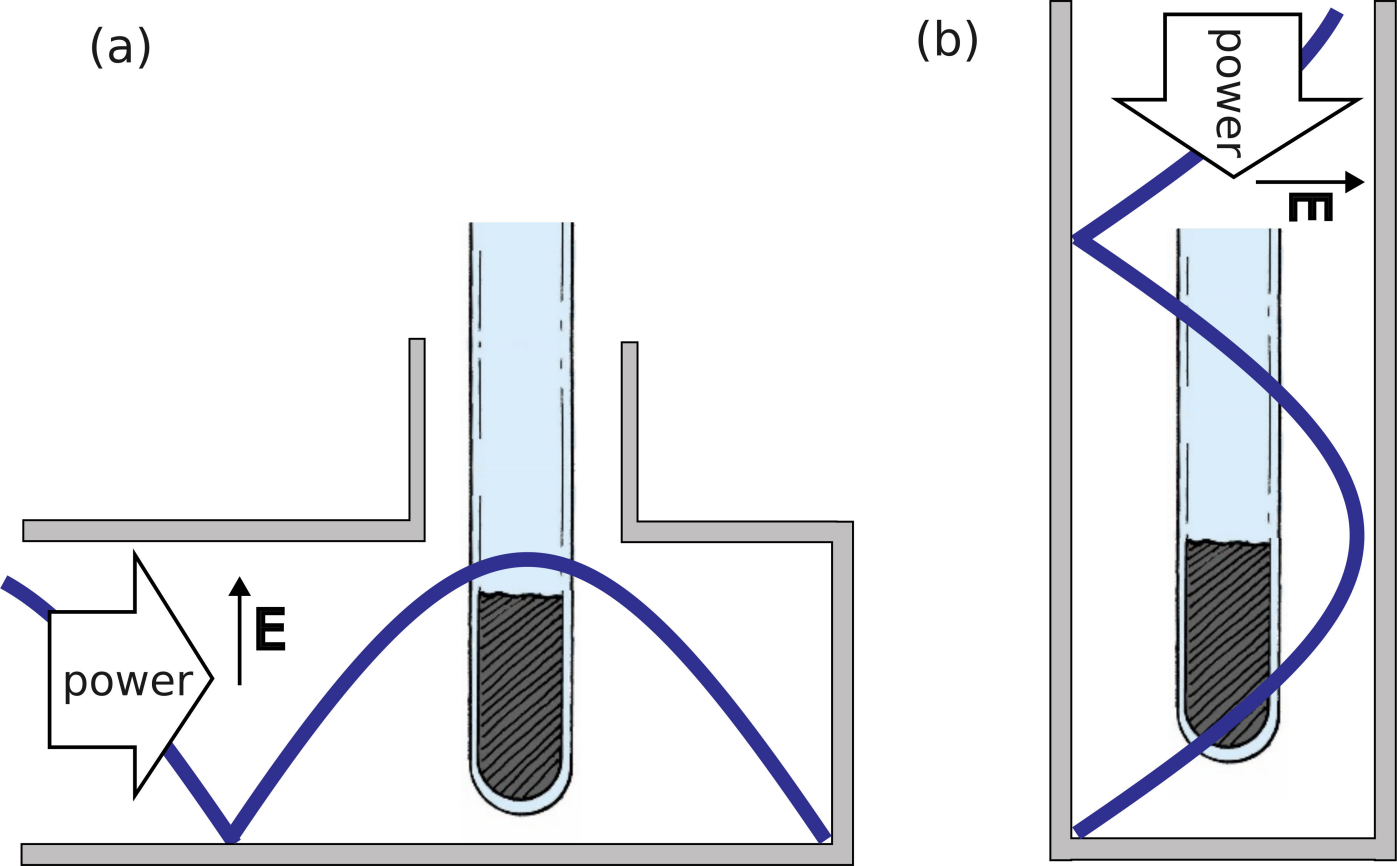
Illustrations of dielectric samples in a TE_10_ WG standing wave mode (a) perpendicular sample loading (b) parallel sample loading.

Constrained by the design of the applicator and choke, each mixture of powdered starting materials was contained in a sealed cylindrical silica ampoule with the length of the tube oriented perpendicular to the direction of MW propagation along the WG (figure 2*a*). This leads to significantly increased efficiency of power absorption and uniformity of heating compared with the alternative parallel orientation (figure 2b) and results in more reliable syntheses [14,29,30]. The parallel orientation suffers from the need for either additional dielectric filling or an adjustable resonant iris coupling [31]. As described in the electronic supplementary material, the WG is set up in TE_10_ mode, where the sliding short-circuit controls the position of the standing wave to maximize coupling to the sample.

**Figure 3.**
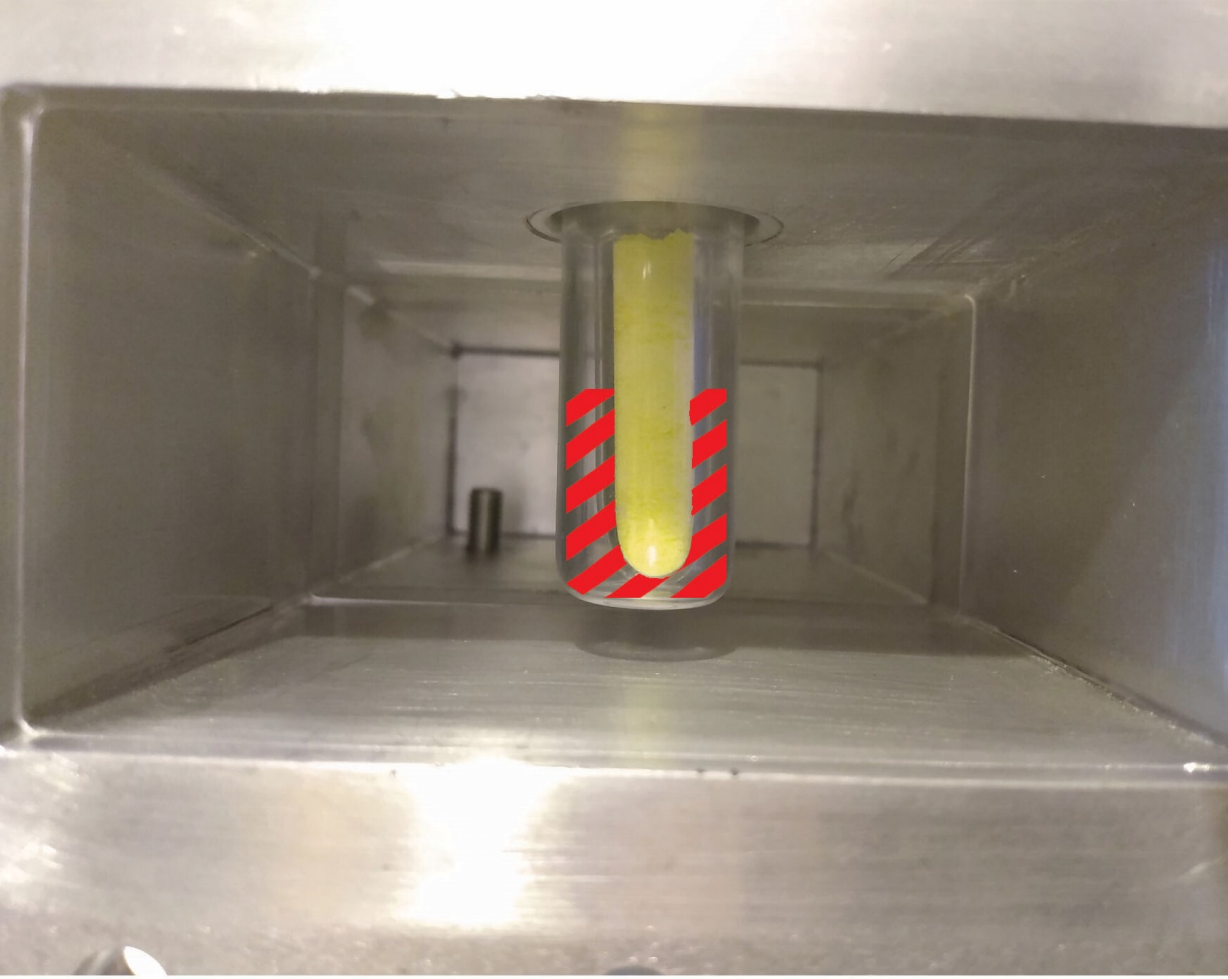
Photograph/schematic of sample loading within the applicator WG section. An ampoule (here containing yellow sulfur powder) is situated in a silica support tube. A fixed bed of graphite powder (shown as hatched red lines) acting as a susceptor can be placed around the ampoule within the support tube.

Nevertheless, the reaction of some samples failed to initiate within a period of several minutes and for these experiments the induction period was reduced by the introduction of a suitable MW susceptor. In these cases, the sealed ampoules were placed into a larger-bore silica tube to which an approximately 5 mm depth of graphite powder could be added (figure 3).

The penetration depth or skin depth (*δ*_*s*_) (also known as the attenuation distance), is a function of applied frequency (*f*), bulk resistivity (*ρ*, or conductivity, *σ*) and relative permeability (*µ*_*R*_) that determines the extent of wave propagation in a substance (such as a conductor) [31–33]. The value of *δ*_*s*_, therefore, increases with increasing *ρ* (decreasing σ) and decreasing *µ*_*R*_ (and decreasing *f*) as shown in equation (2.1). Graphite has reported *σ* and *µ*_*R*_ of 1 × 10^5^ Sm^−1^ and 1 Hm^−1^, respectively, corresponding to *δ*_*s*_ = 29 µm, meaning that micron-size particles can be readily heated in a MW field [33]:


(2.1) $$\delta_{s}=\sqrt{\frac{2\rho }{2\pi f\mu_{0}\mu_{R}}}.$$

**Figure 4.**
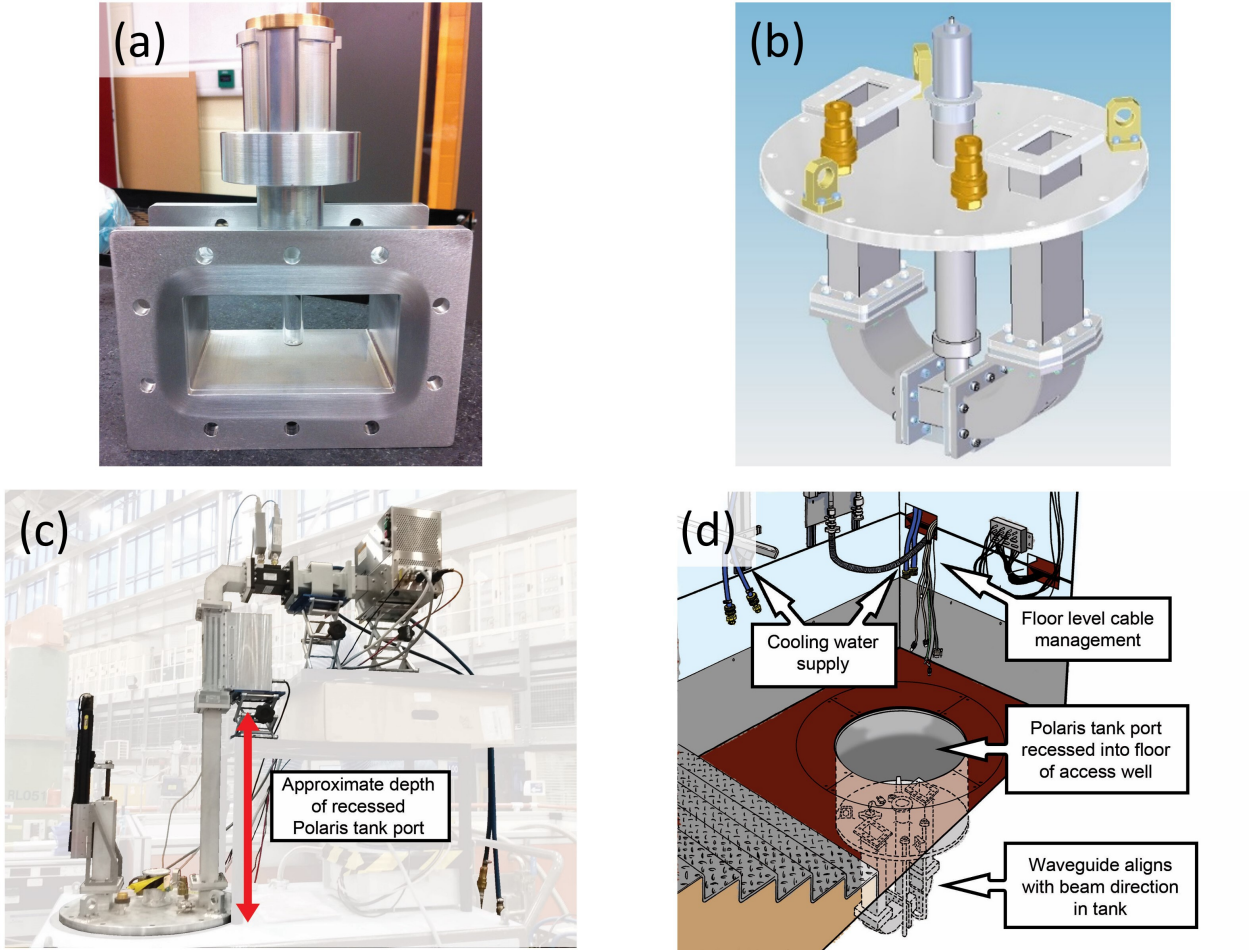
(a) Silica support tube suspended in the centre of the sample applicator section of the WG, held in place by the aluminium support block; (b) schematic of the U-shaped return bend applicator affixed to a Tomkinson flange with the sample stick in place; (c) *in situ* reactor configuration with WG and MW components in position; (d) cutaway diagram of Polaris access well showing sample applicator/Tomkinson flange in operational position within the recessed floor aperture.

**Figure 5.**
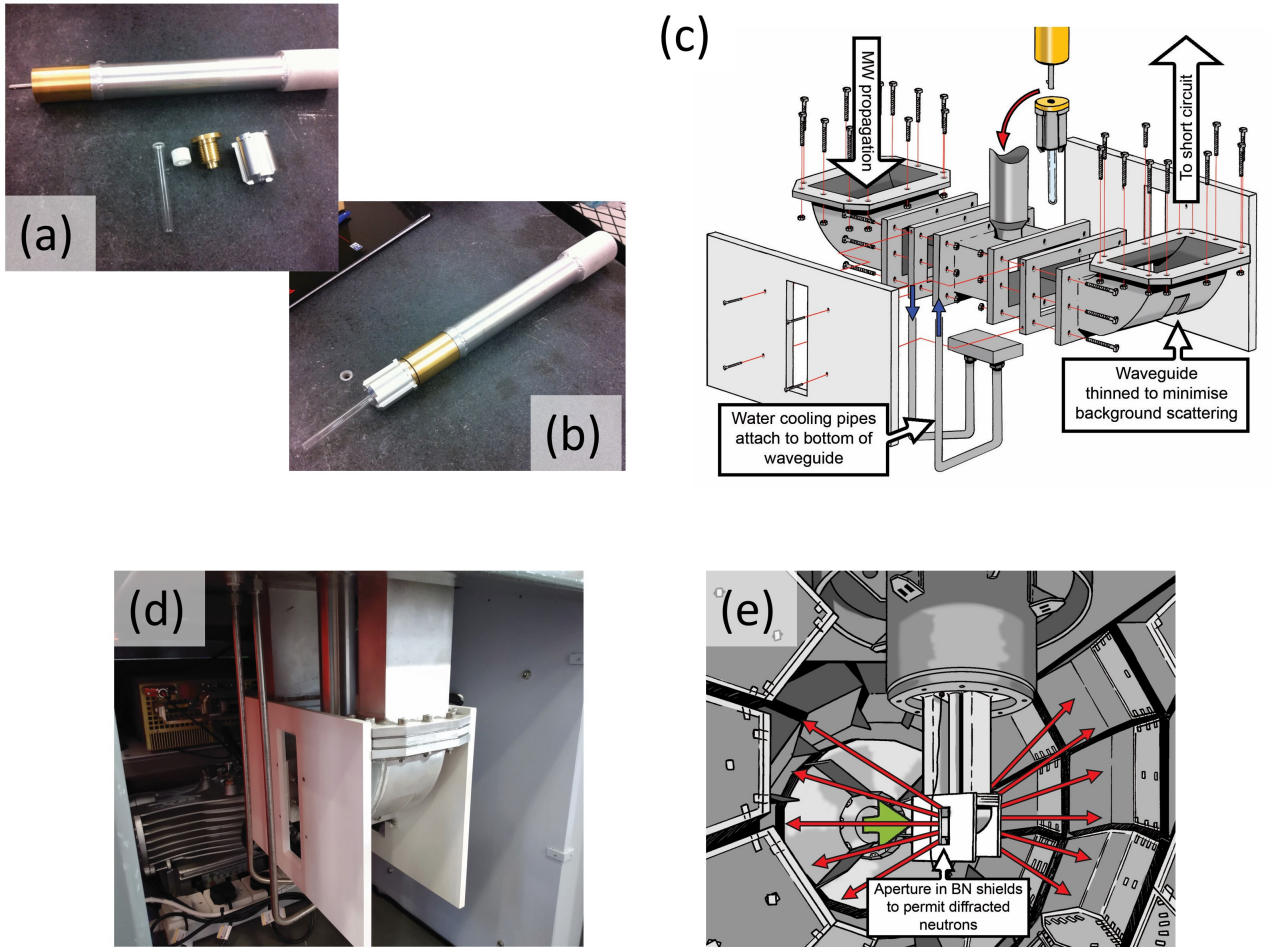
(a) Sample/ampoule support block components and quick-release stick; (b) silica support tube inserted into support block and attached to quick-release stick; (c) exploded diagram of the sample applicator return bend showing the thinned sections of the WG and BN shielding fitted externally; (d) U-shaped sample applicator with rectangular, white BN shielding, aperture and cooling water supply pipes; (e) schematic of the applicator positioned inside the Polaris sample tank, showing the neutron pathway from the beamline (rear of return bend) towards the sample and through the BN shield aperture to the 2*θ* ~ 75−113° (bank 4) detectors.

Typically applying up to 100 W incident power (referred to as forward or applied power) generated sufficient heat to cause the reaction to go to completion. The power that is not absorbed by the load is reflected back along the WG (hence ‘reflected power’), so with measurement of both, the reflected power can be subtracted from the forward power to determine the amount absorbed by the sample. A Dataq Instruments DI-1100 4-channel USB Data Acquisition (DAQ) device was installed for this purpose. By converting the difference between voltages across four-pin outputs on the dual power metre, the associated WinDAQ software could record both the forward and reflected MW power in real time. Although a graphite susceptor was initially considered necessary for some syntheses (see §3), the DAQ was used subsequently to tune the short circuit for maximum sample coupling. From the DAQ data, it was also possible to determine the end of the reaction induction period prior to accelerated heating more precisely (allowing an appreciation of the duration of maximum MW absorption and associated heating). These lab-based experiments paved the way for samples to be heated *in situ* in a neutron beam *without the aid of a susceptor*. To operate the SMC reactor within the Polaris neutron diffractometer required a number of modifications to the benchtop configuration. Most significantly the WG was required to accommodate the sample position in the incident neutron beam, which is located within a large vacuum enclosure (see §2c below). This necessitated the design of a ‘U’-shaped WG such that only the applicator/choke component containing the sample was positioned inside the vacuum tank (figure 4). The walls of the aluminium WG were thinned over a section matching the neutron beam cross-section at the curves of the U-shaped return bend (i.e. where the beam enters and exits the WG) to minimize both the attenuation of the incident beam and the background scattering from the WG walls (figure 5c). Furthermore, because Polaris operates under vacuum, while MW apparatus normally operates in air, all components located inside the beamline sample tank had to be air-tight. This was achieved either by fitting graphite gaskets between the components during assembly or by welding them together.

The silica-tube reaction vessel is located in the path of the neutron beam at the lowest point of the return bend. The tube is held in place using an aluminium support block (figure 5a) that is inserted with a quick-release stick (figure 5b) to rest at the base of an approximately 400 mm long cylindrical access tube, which extends up to the exterior of the vacuum tank. This vertical shaft allows a sample to be inserted and removed using the stick incorporating a quick-release mechanism; details of the support block and insertion mechanism are included in the electronic supplementary material. Neutron-absorbing boron nitride (BN) shields were affixed to each side of the lower section of the sample applicator to prevent neutrons scattered by the aluminium WG from reaching the detectors (figure 5c,d). A rectangular aperture in the BN shields then ensured diffraction patterns collected by the Polaris 2*θ* ~ 75−113° detectors contain Bragg reflections exclusively from the sample; (figure 5e). The configuration of all these components to operate within the Polaris sample tank, ruled out the inclusion of an infrared thermometer or probe to record changes in sample temperature, as was possible with the benchtop set-up. Placing a probe within the WG would interfere with MWs and line-of-sight from above would be blocked by the sample support block. Due to the neutron path, an infrared thermometer could not be set up at either 90° curve of the WG. Similarly, the BN shield and gap for diffracted neutrons blocked access from either side. Finally, the bottom face was ruled out as this was the location where the water-cooling heat exchanger plate and WG thermocouple were connected.

A modification to the short-circuit mechanism was required for the SMC reactor to be operated on Polaris due to restricted access. In the laboratory, the position of the sliding short-circuit plate is adjusted manually using a precision screw drive mechanism. The safety-limited operator access on Polaris dictates that the short circuit is operated remotely through a motorized track attached to the extended plunger through a bespoke bracket, as described in the electronic supplementary material. The electronic control was essential to allow the standing wave to be adjusted from outside the Polaris access well at the same workstation that the MW power controls were set.

### (b) Sample preparation and preliminary characterization

All materials were synthesized from stoichiometric mixtures of the respective elemental powders: Sb (Aldrich, 99.5%), Bi (Thermo Scientific, 99.5% or Alfa Aesar, 99.999%) Se (Alfa Aesar, > 99%) and Te (Sigma Aldrich, 99.997%) to prepare product masses of 1 g for initial benchtop test experiments or 5 g for subsequent scale-up experiments and *in situ* SMC reactor commissioning experiments; the purpose of utilizing 5 g samples was to maximize the amount of sample in the neutron beam. Samples of Bi_2_X_3_ for *in situ* experiments were prepared inside a nitrogen-filed MBraun LABstar pro recirculating glovebox (H_2_O and O_2_ levels ≤ 5 ppm). Ground powders were transferred into an 8 mm bore, 12 mm outer diameter silica tube (pre-sealed at one end) that had been cut to the appropriate length for each SMC reactor configuration. A long-stem funnel was used during sample loading to limit material adhesion to the tube walls. Evacuated tubes (approx. 10^–6^ mbar) were sealed using a natural gas/oxygen blow torch (max. *T* ~2770℃) to evenly heat the silica, which has a softening temperature of approximately 1600℃ [34]. Laboratory SMC samples were typically irradiated for times between 20 and 180 s. *In situ,* SMC samples were irradiated for times of approximately 60, 90 or 120 s (each determined precisely). Further details of the heating regimes are presented in §3. Powder X-ray diffraction data were collected using a PANalytical X’Pert Pro MPD diffractometer in Bragg–Brentano reflection geometry. A 1 mm divergence slit was used and collection parameters were specified for a 2*θ* range of 5−85° with an angular step size of 0.01671° collected for a duration of 16 min at a step rate of 0.047074° s^−1^. The resulting .xrdml data files obtained were used for phase identification and converted to .gsas file format using the PowDLL file converter package for subsequent Rietveld refinement to obtain phase fractions [35].

### (c) The Polaris neutron diffractometer

**Figure 6.**
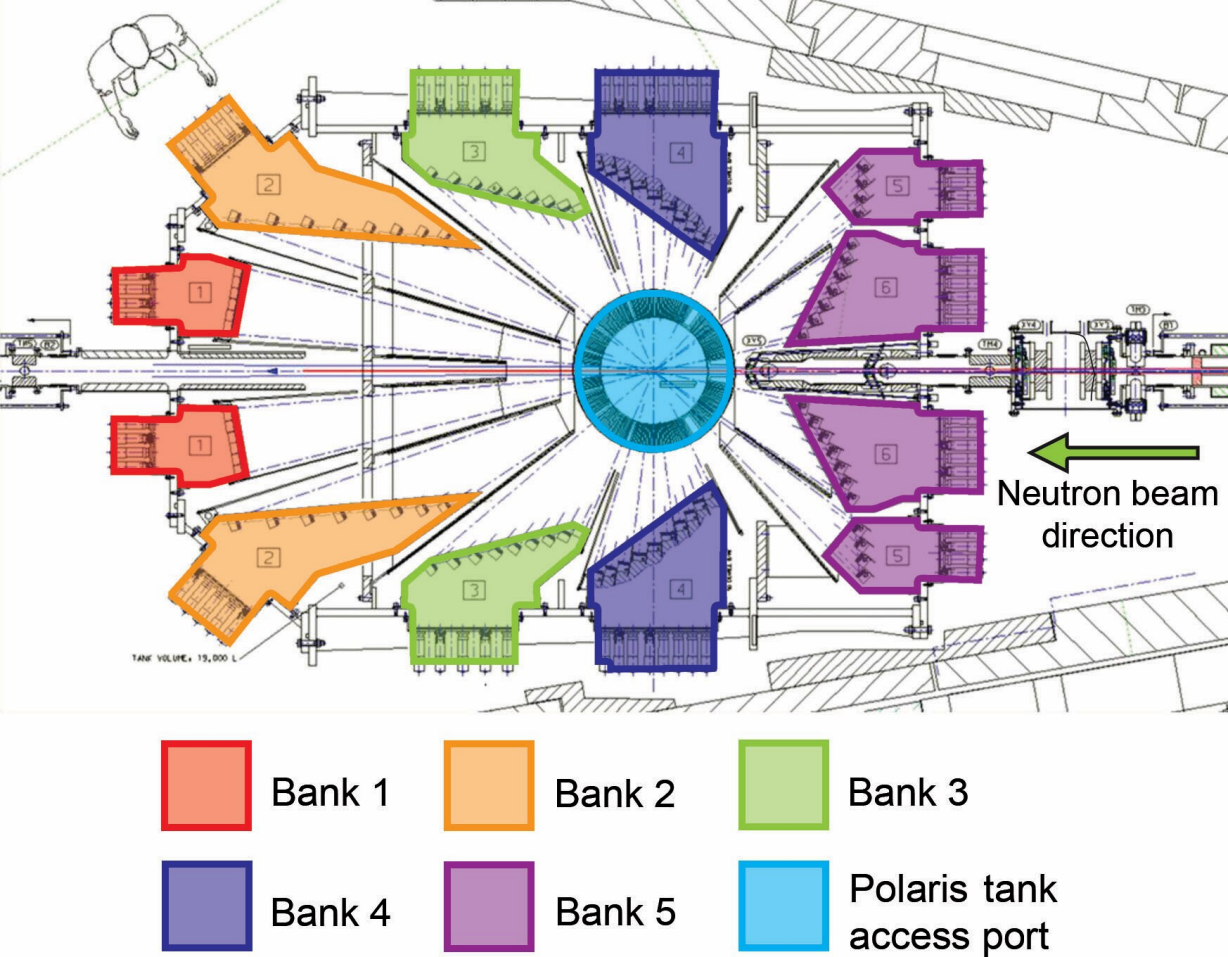
Schematic of the Polaris diffractometer highlighting the five detector banks in relation to the tank access port. The neutron beam enters the sample tank from the right, through the centre of the bank 5 detectors [31].

Time-of-flight (ToF) PND data were collected on the Polaris medium resolution ToF diffractometer at the ISIS Pulsed Neutron and Muon Source, Rutherford Appleton Laboratory, UK [36]. With a relatively short primary flight path of 14 m Polaris receives a high flux of incident neutrons and this combined with a large detector solid angle coverage of almost 2π ster enables the instrument to collect datasets very quickly (as short as a few tens of seconds in the most favourable cases). These features emphasize the exceptional capability of Polaris for performing time-resolved studies. Viewing an ambient temperature water moderator, the incident beam is rich in short wavelength neutrons enabling neutron diffraction patterns to be collected to very low *d*-spacings (approx. 0.3 Å), which is extremely beneficial for the accurate refinement of site occupancies and atomic thermal displacement parameters in disordered materials. The detectors on Polaris are grouped into five separate banks, each covering a discrete 2*θ* range (figure 6). The detectors at low angles access the longest *d*-spacings (*d*_max_ ~ 40 Å), but with the lowest resolution (∆d/d ~ 2.5 %), whereas those at the highest 2*θ* angles have superior resolution (∆d/d ~ 0.3 %) but only access a maximum d-spacing of approximately 2.7 Å. As discussed above, the SMC MW reactor incorporates BN shields that enable diffraction patterns collected in detector bank 4 (2*θ* ~ 75−113° and *d*_max_ ~ 3.7 Å) to be free from background Bragg scattering from the beam entry/exit windows in the aluminium WG. Access to the Polaris sample position is through a 400 mm diameter aperture (highlighted in blue in figure 6) through which the U-shaped return bend WG and sample applicator section of the SMC reactor is lowered using a beamline crane.

### (d) ToF PND data collection

The height of the incident neutron beam was reduced to approximately 20 mm during data collection to ensure the natural divergence of the incident beam penumbra did not cause it to strike the top or bottom of the WG and also to minimize the amount of background scattering from the silica sample tube. In addition, datasets were collected from empty silica tubes in the SMC reactor so that the structured background arising from the reaction vessel could be subtracted from the experimental data. For reference, an initial diffraction pattern was collected from the reaction mixture in the SMC reactor before initiating MW heating. A similar procedure followed at the conclusion of MW heating and sample cooling to allow verification of successful synthesis. Both these initial and final datasets were collected with the *Ibex* instrument control software operating in Histogram mode. In this mode, the ToF values measured are the times taken between the neutron pulses being created in the ISIS target and the scattered neutrons reaching the detectors. Every 20 ms, as successive pulses of neutrons are generated, the timing clock is reset to zero and the pattern is produced by accumulating (histogramming) counts from a large number of neutron pulses into a set of discrete time channels. The process continues until the time channels contain sufficient counts that the diffraction pattern is of the desired statistical quality [37,38]. Significantly, when studying chemical processes, there is a ‘dead’ time of approximately 15 s between completing the collection of any diffraction pattern and starting the next one while the dataset is written to file and the histogram memory cleared. Also, if a reaction proceeds quickly enough that there are significant changes in the diffraction pattern during the course of its data collection there is no way of determining what those changes were; nor when they occurred.

Therefore, an alternative strategy, named Event Mode, was used for *in situ* measurements while the MW power was switched on, and also continuing for a period of 1−2 min after the MWs had been switched off, during which rapid changes in the sample composition were taking place. In this mode, *Ibex* uses a Real Time Clock to record the absolute date and time that the detected neutron events occurred at the detectors into a single extended dataset, rather than collecting a series of individual diffraction patterns over the course of an experiment, thereby avoiding any periods of dead time [37,38]. Importantly with this procedure, each experimental dataset, collected over a period of approximately 5−10 min during the MW-induced reaction, could subsequently be split into any number of time slices of arbitrary duration after the experiment had finished, with the number and duration of the time slices chosen to optimize the compromise between good time resolution (short time slices) and good counting statistics (large number of counts). This means that if the data for an experiment lasting 3 min were sliced into nine equal sections, each slice would represent a discrete collection of neutron events within that specific 20 s period of time. The same data could be sliced into 5 s sections creating a neutron timeline of 36 × 5 s chunks. Also, because a MW absorption profile was collected at the same time as the neutron scattering data, the use of Event Mode during the reactions allowed us to correlate features in the absorption profile with changes in the diffraction patterns. This absorption profile was determined using the forward and reflected MW power values recorded in real time by the DI-1100 DAQ instrument. All data normalization and post-experiment time-slicing was performed using the *Mantid* neutron scattering and muon spectroscopy data processing and visualization software [39]. Note that while only data collected in Polaris detector bank 4 are reported here, the geometry of the SMC MW reactor allows diffraction data to be collected in the low- and high-angle detector banks, albeit with significant background peaks from the aluminium Bragg reflections. The pre- and post-reaction diffraction patterns were analysed by the Rietveld method using the *General Structure Analysis System* (*GSAS*) software package primarily to identify and quantitatively determine the respective phase compositions [40,41]. In addition, structure refinements of phases from selected time slices extracted from the Event Mode datasets could also performed. However, this was limited to diffraction patterns that were of sufficient statistical quality that stable refinements could be achieved.

## 3. Results and discussion

### (a) Sb_2_Se_3_ and Sb_2_Te_3_ benchtop SMC reactor experiments

Both approximately 1 and approximately 5 g samples of Sb_2_X_3_ (X = Se, Te) were synthesized from the elements according to equation (3.1):


(3.1) $$2Sb+3X\to Sb_{2}X_{3}.$$

Successful synthesis of both Sb_2_Se_3_ and Sb_2_Te_3_ had been previously reported using an MMC MW (1000 W CEM MDS 2100) reactor [14]. These prior studies found that phase pure Sb_2_Te_3_ required multiple heating cycles, an approach that we wanted to avoid in the design of *in situ* experiments for Polaris. The first batch of approximately 1 g samples was heated using 600 W forward power, with the synthesis duration marked by the observation of a characteristic blue glow from arcing/plasma formation in the sample applicator. The heating was allowed to progress for 20 s after the first observation of this glow. The progress of these reactions and product purity was inconsistent. Only one of several Sb_2_Se_3_ experimental runs progressed to completion to yield products of high purity. Rietveld structure refinement against PXRD data demonstrated the presence of orthorhombic (Space group *Pnma*) Sb_2_Se_3_ with a phase fraction of 96.4 (1) wt%. The remaining reflections were attributable to the remaining Sb (electronic supplementary material). Apparently, proportions of Se were vaporized on heating and deposited on the ampoule walls, not participating in the reaction with Sb.

**Figure 7.**
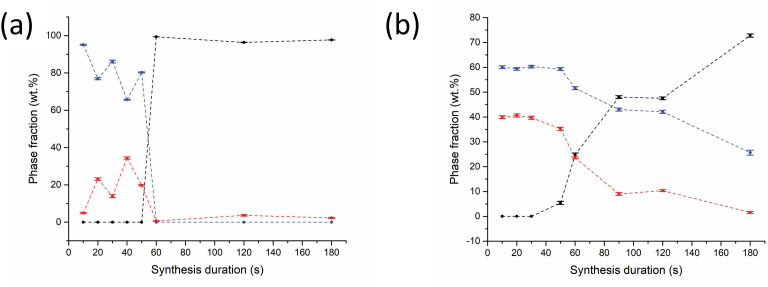
Plots of relative phase fractions against synthesis duration using 100 W forward power with the aid of a graphite susceptor for (a) black datapoints - Sb_2_Se_3_ (*R*$\bar{3}$*m*), red datapoints - Sb (*R*$\bar{3}$*m*) and blue datapoints - Se, (b) black datapoints - Sb_2_Te_3_ (*R*$\bar{3}$*m*), red datapoints - Sb (*R*$\bar{3}$*m*) blue datapoints; -Te (*P3_1_21*). The dotted trend lines serve as a guide to the eye.

All further Sb_2_X_3_ lab SMC samples were heated under reduced forward power (100 W) but with the aid of a graphite susceptor to facilitate synthesis within a reasonable induction period and to minimize potential hot spots. Under these conditions, synthesis times of less than 60 s were not sufficient for accelerated heating to occur. Times greater than 60 s consistently yielded Sb_2_Se_3_ phase fractions greater than 96% (figure 7a). It was evident that once an optimum heating duration was achieved, there was negligible further improvement in phase purity with time. Under these conditions, a plateau in the phase fraction with time is achieved and it can be assumed that all available Se has been consumed. Following the results of the Sb_2_Se_3_ experiments, Sb_2_Te_3_ MW syntheses were performed under similar conditions; at 100 W forward power using a graphite susceptor (figure 7b). Telluride samples heated for less than 50 s resulted in no reaction. For heating times greater or equal to 50 s, aside from the starting materials, trigonal (*R*$\bar{3}$*m*) Sb_2_Te_3_ is the only product and the phase fraction increases with synthesis duration from 5.4 (6) wt% at approximately 45 s to 72.8 (7) wt% at 180 s.

Some of the variability in the phase composition of unreacted starting materials (most notable in the Sb_2_Se_3_ sample set) are probably due to the inhomogeneity of the recovered powders (where some reactants are probably displaced or change state after heating). Following the successful synthesis of Sb_2_Te_3_, the trend in phase fraction with time suggests that still longer heating durations could achieve even greater levels of phase purity. At the time, further longer duration syntheses were curtailed in favour of attempts to scale up beyond 1 g for *in situ* experiments while in parallel striving to achieve successful partial substitution at the chalcogen site in a series of Sb_2_Se_3−*x*_Te_*x*_ solid solution syntheses as part of a wider investigation. Longer duration Sb_2_Te_3_ syntheses have since been investigated while MW preparations of approximately 5 g mixed chalcogen samples Sb_2_Se_3−*x*_Te_*x*_ showed extremely promising results for 0 < *x* ≤ 1. These experiments and materials will be discussed in a future publication.

### (b) *In situ* neutron diffraction studies of Sb_2_Se_3_ synthesis

**Figure 8.**
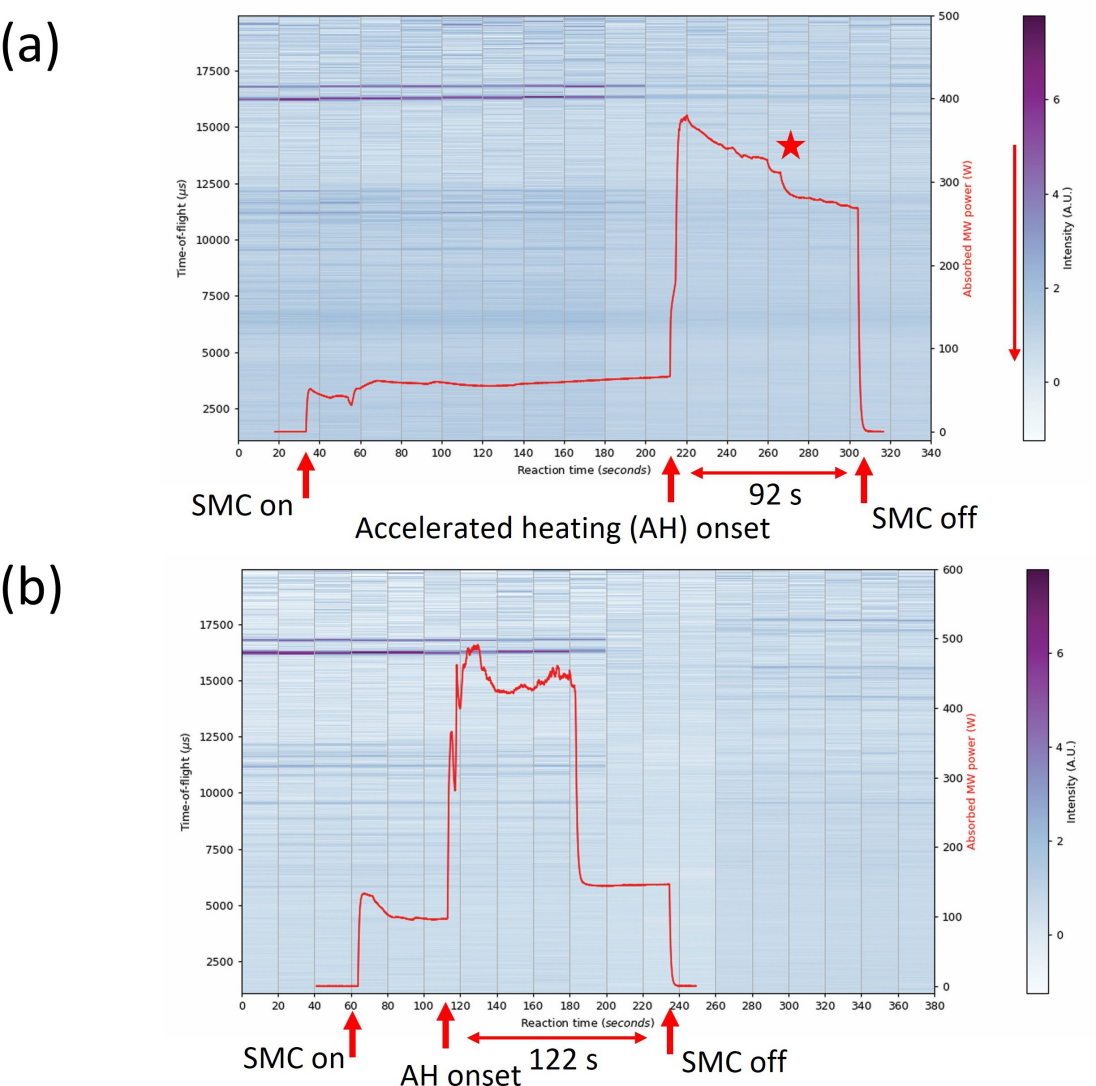
Contour colourmap plots of event mode TOF data (shades from blue to purple) on the left y-axis and absorbed MW power profile (red datapoints) on the right y-axis as a function of 20 s slices over the full experiment duration using 500 W forward power: (a) 92 s synthesis of Sb_2_Se_3_—the red star indicates the onset of a new phase formation at 56 s; (b) 122 s synthesis of Sb_2_Se_3_. The intensity of the diffraction peaks is signified by the colour histogram on the right of each plot.

Two 5 g samples of 2 : 3 Sb : Se were prepared to probe the effects of extended heating on phase composition and reaction pathway. The samples were heated for either 92 or 122 s from the onset of accelerated heating (figure 8). With the use of the DAQ to tune the short circuit for optimal sample coupling in the SMC *in situ* reactor, it was expected that synthesis could be achieved *without* the aid of a graphite susceptor. Apart from removing graphite (reflections) from the system (diffractograms), susceptor-less heating would allow the power profiles to be closely correlated with events intrinsic to the reaction components in the powder profiles. For example, a more accurate record of the onset of accelerated heating for each reaction mixture could be obtained by eliminating the effects of the initial coupling with graphite. The step change in the calculated power absorption profile between high and low plateaux of MW absorption initially seemed to indicate new phase formation from approximately 55 s into the accelerated heating period (figure 8a). These features are not very clear but do demonstrate the weakening of the intensity of starting material peaks in the 20−25 s period prior to accelerated heating. Bragg reflections indicative of Sb_2_Se_3_ being formed do start to appear far into the synthesis, becoming more prominent after the MW power was switched off, suggesting that the products are in the process of forming. The two plateaux and the drop in absorbed power between them, particularly pronounced in the 122 s synthesis (figure 8b), suggest a significant drop in *ε*′ implying that the load has changed, i.e. the reaction has concluded, or at least that the starting materials have been completely consumed.

**Figure 9.**
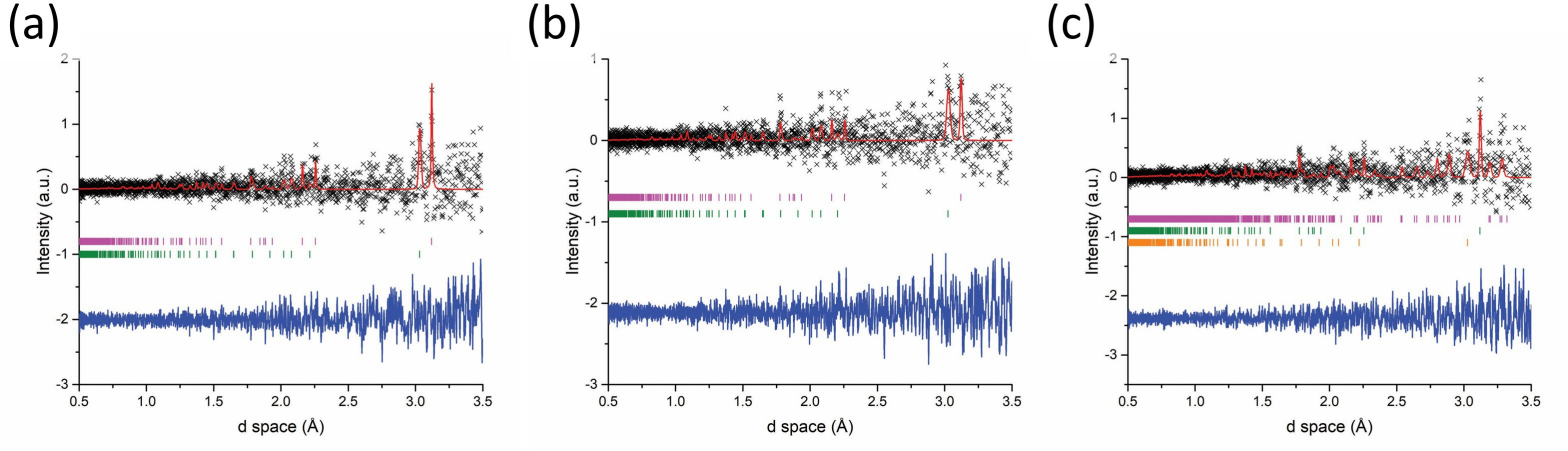
Rietveld fits to diffraction patterns generated from Event Mode ToF data collected during Sb_2_Se_3_ synthesis using 500 W forward power for 92 s. Each dataset contains only those neutrons counted within the specified 20 s period of time during the reaction. Key: x - observed data, red plot - calculated fit, blue plot - difference profile: (a) 60−80 s after accelerated heating—Bragg reflection markers: magneta markers - Sb (*R*$\bar{3}$*m*), green markers - Se (*P3_1_21*) (b) 80−100 s after accelerated heating—Bragg reflection markers: magenta markers - Sb (*R*$\bar{3}$*m*), green markers - Se (*P3_1_21*); (c) 100−120 s after accelerated heating—Bragg reflection markers: magenta markers - Sb_2_Se_3_ (*Pnma*), green markers - Sb (*R*$\bar{3}$*m*), range markers - Se (*P3_1_21*).

The event mode data for the 92 s synthesis are represented by the set of 20 s slices shown in figure 9. While it was possible to index the starting materials from early timepoints in the *in situ* data and observe reduced intensity of their distinctive peaks as heating progressed (figure 9a,b), the very poor signal : noise ratio and low resolution made definitive identification of product phases and their associated phase fractions with errors difficult. In the 100−120 s after accelerated heating (figure 9c) some intense peaks, particularly at low d spacing, were able to be fitted to Sb_2_Se_3_ appearing for the first time and its phase fraction calculated to be 43 (3) wt%. It is possible that Sb_2_Se_3_ may form earlier but the most prominent peaks could be overlapped or subsumed by observed points in the same d-spacing region. The diffraction lines became more intense and much clearer after the MW power was removed. This could indicate that Sb_2_Se_3_ crystallizes on cooling from an amorphous or melt phase. This scenario is supported not only by the noisiness of the data but also from the knowledge that Sb melts at 630℃ and Se at 220℃ and that the reaction temperature is likely to exceed those thresholds [42]. Unfortunately, temperature measurements were not recorded for the Sb_2_Se_3_ synthesis experiments, but other experimental samples run in the benchtop SMC reactor were seen to reach typical temperatures of 700−800℃ under similar conditions. Taking a series of temperature profile measurements and correlating with power absorption data for Sb_2_X_3_ samples in subsequent *in situ* experiments should clarify this behaviour.

The *in situ* data could also be affected by the volatile behaviour of the reactants during heating with the solid phases being either displaced or melted. This appears to be especially relevant for Sb, which is reduced from 72 (3) to 42 (3) wt% in the 60—100 s time slice in the period after accelerated heating. Melting would result in diffraction patterns consisting of much broader/diffuse features from amorphous phases that only begin to crystallize and produce sharper Bragg reflections on cooling. (Whereas if quantities of samples were displaced out of the beam cross-section then the background would increase correspondingly.)

**Figure 10.**
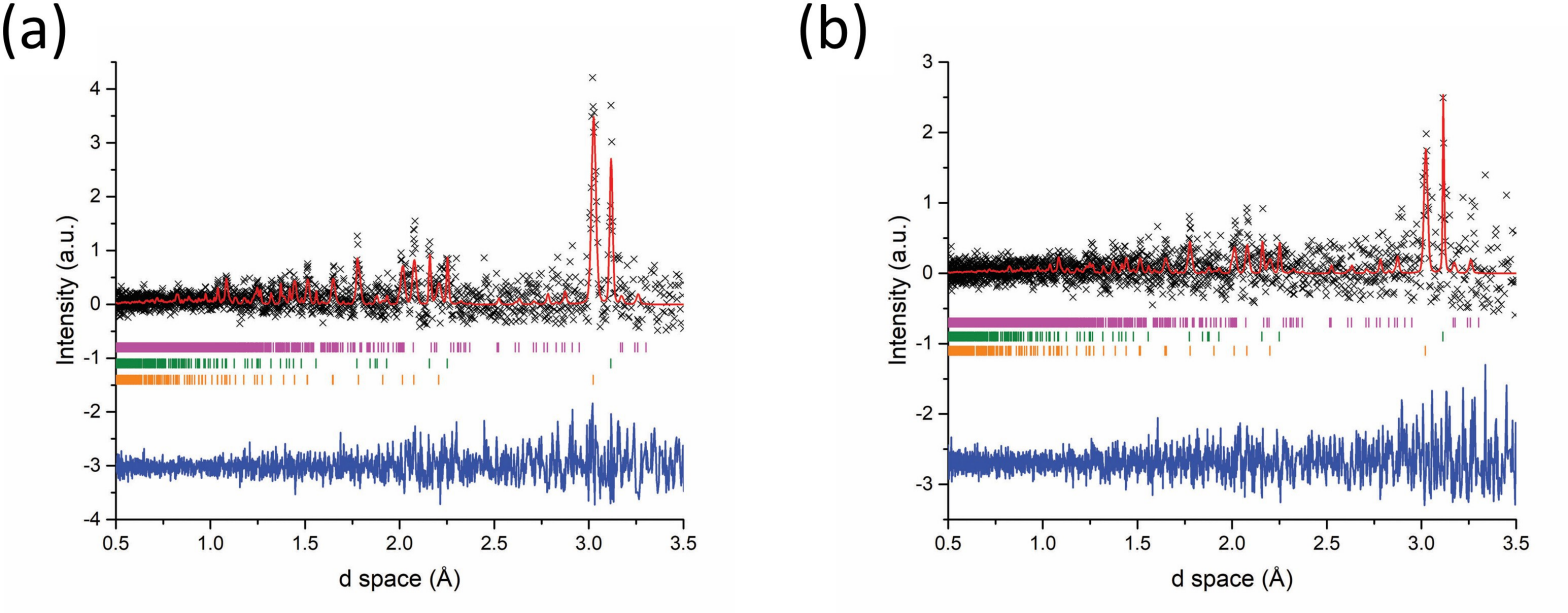
Rietveld fits to diffraction patterns generated from Event Mode ToF data collected during Sb_2_Se_3_ synthesis using 500 W forward power for 122 s. Each dataset contains only those neutrons counted within the specified 20 s period of time during the reaction: (a) 60−80 s after accelerated heating (b) 80−100 s after accelerated heating; Key: x - observed data, red plot - calculated fit, blue plot - difference profile, Bragg reflection markers: magenta markers - Sb_2_Se_3_ (*Pnma*), green markers - Sb (*R*$\bar{3}$*m*), orange markers - Se (*P3_1_21*).

Despite the low signal : noise ratio, the *101* peak for Se at d ≈ 3 Å is much clearer in the 122 s synthesis sample versus the 92 s sample (figure 10a). The peak progressively reduces in intensity over the following 20 s of sliced data (figure 10b) as the Se is consumed in the reaction. These observations further demonstrate that the volatility and/or melting of the material(s) poses a significant challenge to *in situ* characterization. The use of a parametric Rietveld refinement method, such as has been proposed by Stinton and Evans, which enables the fitting to an evolving structural model as a function of time, may assist in addressing this challenge in future studies [43]. However, changing the refinement approach cannot fundamentally improve the data quality and extending the neutron collection window by several minutes into the sample cooling period combined with gentler heating with reduced forward power (increasing the induction period) should improve the value of the experimental outcomes. Nevertheless, a success of the experimental configuration proved to be the ability to produce Sb_2_X_3_ samples without introducing multiple MW-heating cycles. Furthermore, the MW susceptor required in benchtop experiments could be dispensed with without adversely affecting the short induction period required for *in situ* experiments.

**Figure 11.**
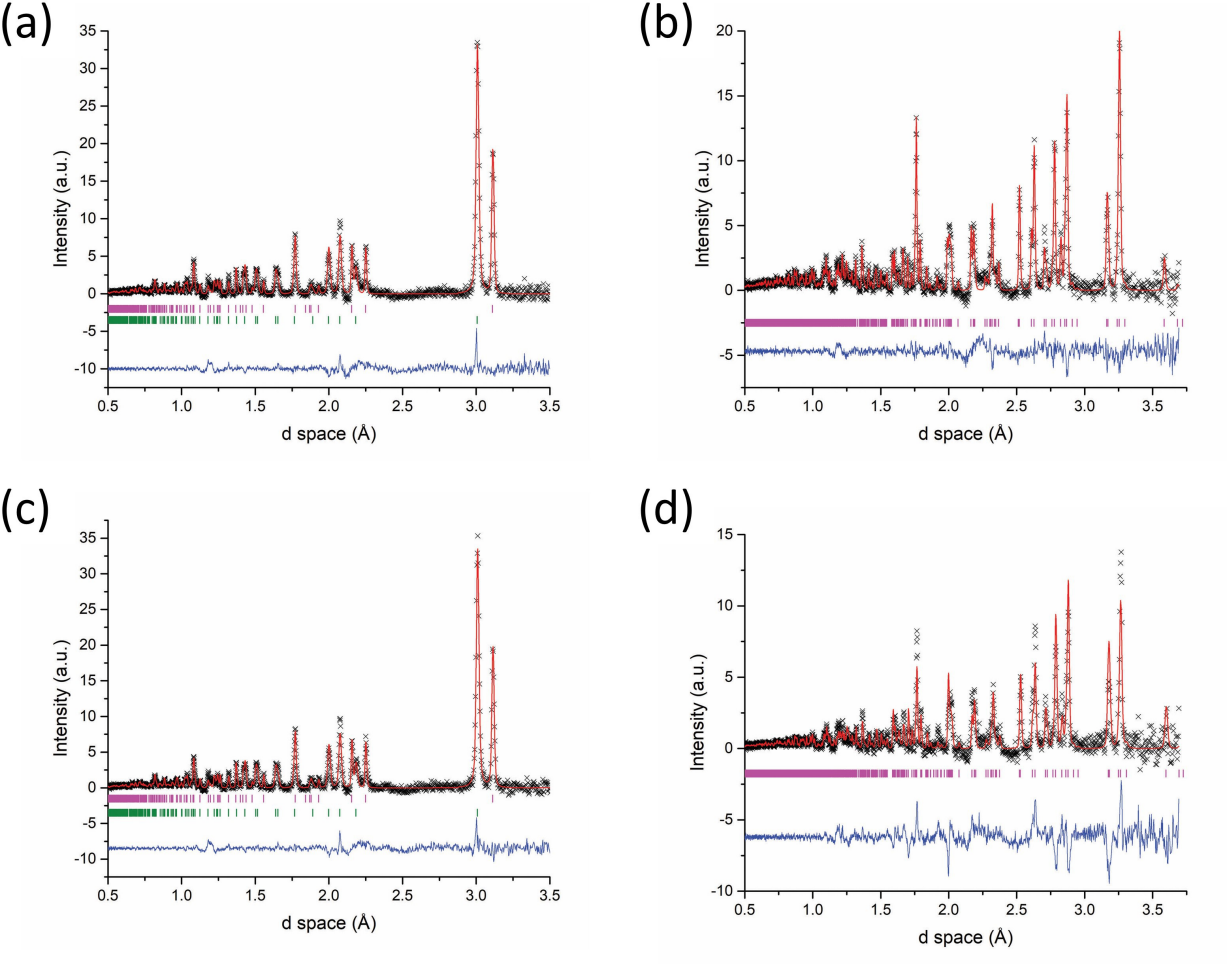
Rietveld fit to histogram mode data collected from Sb_2_Se_3_ synthesized using 500 W forward power. Each dataset was collected for a period of 5 min either immediately before active MW heating or after the reaction had completed. Key: x -observed data, red plot - calculated fit, blue plot - difference profile (a) pre-92 s reaction starting materials—Bragg reflection markers: magenta markers - Sb (*R*$\bar{3}$*m*), green markers - Se (*P3_1_21*); (b) post 92 s synthesis—Bragg reflection markers - magenta markers - Sb_2_Se_3_ (*Pnma*); (c) pre-122 s synthesis starting materials—Bragg reflection markers - magenta markers - Sb (*R*$\bar{3}$*m*), green markers; Se (*P3_1_21*); (d) post 122 s synthesis—Bragg reflection markers: magenta markers - Sb_2_Se_3_ (*Pnma*).

Unfortunately, the quality of the short-duration time slice patterns extracted from the Event Mode data is not currently sufficient to allow the chemical reaction dynamics to be followed and is too poor for meaningful structure analysis. Similar inherent restrictions are likely to apply to many chemical systems, especially those containing solid reactants with relatively low melting (and boiling) points. However, it should be possible to modify *in situ* experiments to obtain data allowing greater insight and understanding. Although tailoring *in situ* reactions by applying a reduced forward power of approximately 200−300 W over expected longer reaction durations—initially trialling such reactions offline—would probably significantly extend the induction times, it may allow the reaction pathway to be more closely examined over an extended accelerated heating period. Although requiring an alternative MW source, altering the incident MW frequency and increasing the penetration depth, δs (equation (2.1)), could allow larger masses of samples to be used, improving counting statistics. Fortunately, the powder patterns obtained from the pre- and post-reaction histograms were of a far superior quality to the *in situ* data and the respective reactant and product phases were easily characterized (figure 11). Both the 92 and 122 s reactions yielded single-phase orthorhombic Sb_2_Se_3_ after cooling.

### (c) Bi_2_Se_3_ and Bi_2_Te_3_ lab SMC reactor experiments

Both approximately 1 g of Bi_2_X_3_ for benchtop experiments and approximately 5 g samples for *in situ* experiments were synthesized according to (3.2):


(3.2) $$2Bi+3X\to Bi_{2}X_{3}.$$

**Figure 12.**
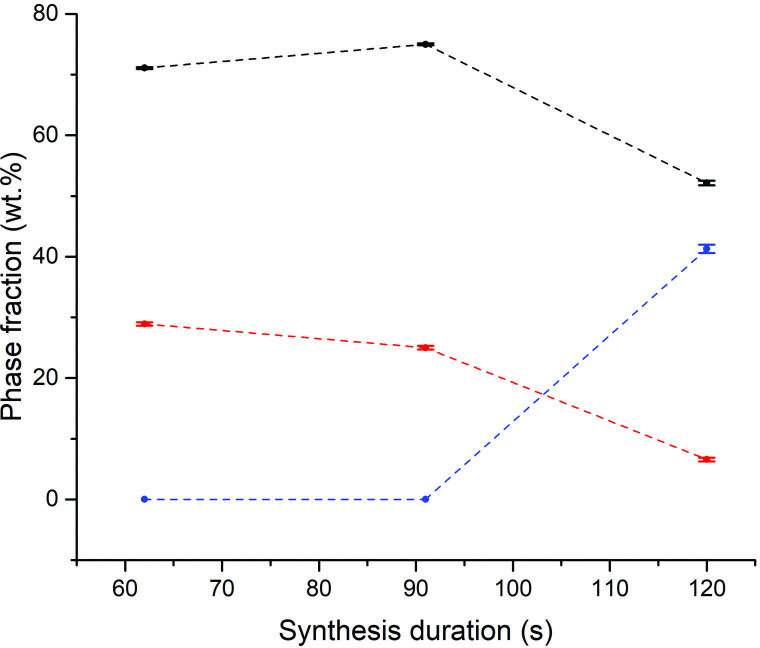
Plots of relative phase fractions against synthesis duration for Bi_2_Se_3_ using 100 W forward power using a graphite susceptor. Key: black datapoints - Bb_2_Se_3_ (*R*$\bar{3}$*m*) red datapoints - Bi_2_SeO_2_ (*I4/mmm*) and blue datapoints - BiSe (*P*$\bar{3}$*m1*). The dotted trend lines serve as a guide to the eye.

Experiments to determine the optimum synthesis conditions were performed in parallel with the Sb_2_X_3_ series of samples, following a similar process. Samples heated for 10 min at 600 W forward power registered only a small amount of MW absorption with no appreciable heating, no physical change in appearance and no plasma formation/arcing observed during the irradiation process. PXRD analysis confirmed no reaction had occurred, so all further samples were heated with the aid of a graphite susceptor. In these experiments, the graphite susceptor was solely responsible for the initial absorption of MW power and it was not possible to identify the point of accelerated heating or a precise synthesis duration from the relatively minor changes in the reflected (absorbed) MW power alone. The total reaction time therefore is relatively approximate on this basis. Given previous precedent from MMC MW experiments performed at a similar scale by Kadhim *et al.* (which required heating times greater or equal to 10 mins interrupted periodically to agitate the sample through shaking), the initial batch of Bi_2_X_3_ samples were prepared using starting materials that were handled in air rather than within an inert glovebox [27]. However, the phase compositions of both Bi_2_X_3_ systems produced using our benchtop MW configuration were discovered to contain several bismuth oxide impurities, including monoclinic (*P12_1_/c1*) Bi_2_O_3_ and tetragonal (*I4/mmm*) Bi_2_O_2_(CO_3_). For the Bi–Se system, tetragonal (*I4/mmm*) Bi_2_SeO_2_ was an additional product whereas for Bi–Te, tetragonal (*P4_1_2_1_2*) TeO_2_ and orthorhombic (*Abm2*) Bi_2_TeO_5_ phases were evident (see electronic supplementary material). The implication was that the Bi powder used was partially oxidized; which was subsequently confirmed by PXRD characterization. Contrasting with the Kadhim *et al.* study, Mastrovito *et al.* reported that the MW synthesis of Bi_2_Se_3_ and Bi_2_Te_3_ required multiple heating cycles using starting materials prepared in an Ar-filled glovebox with excess selenium [14]. Consequently, all our subsequent Bi_2_Se_3_ and Bi_2_Te_3_ samples were prepared using starting materials stored inside an N_2_-filled glovebox (with bismuth verified as phase-pure before use) and were heated for either 60, 90 or 120 s using a graphite susceptor to reduce the reaction induction period. Visual inspection of the Bi–Se samples after heating revealed red deposits on the tube walls above grey ingots as shown in the electronic supplementary material. The Bi–Te samples yielded grey ingots with a metallic lustre along with metallic deposits on the upper tube walls. Despite the further precautions to prevent oxidation noted above, the selenide samples contained a small proportion of Bi_2_SeO_2_, although Bi_2_Se_3_ formed as the majority phase. Intriguingly, peaks corresponding to the trigonal (P$\bar{3}$*m1*) monoselenide, BiSe became visible after heating for 120 s (figure 12) with a corresponding reduction in the other phases, which may be the result of product degradation due to thermal runaway. Alternatively, it is possible that BiSe formed from the reaction of Bi_2_Se_3_ with bismuth oxyselenide, liberating oxygen (or yielding an oxide byproduct). It was hoped that *in situ* neutron experiments might expose the possible origins of such secondary phase formation and provide further details concerning their subsequent fate. Positive results would inform adjustments to future experimental design so that phase-pure Bi_2_Se_3–*x*_Te_*x*_ materials could be produced.

**Figure 13.**
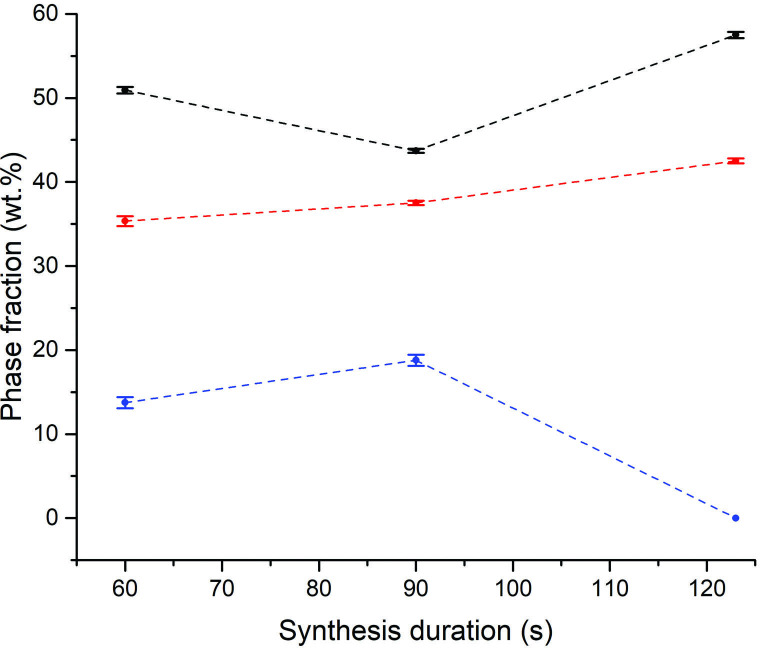
Plots of relative phase fractions against synthesis duration for Bi_2_Te_3_ using 100 W forward power using a graphite susceptor. Key: black datapoints - Bb_2_Te_3_ (*R*$\bar{3}$*m*), red datapoints - Bi_2_TeO_5_ (*Abm2*) and blue datapoints - Te (*P3_1_21*). The dotted trend lines serve as a guide to the eyes.

PXRD analysis of the Bi_2_Te_3_ samples showed that Bi_2_TeO_5_ comprised a significant portion of the phase composition regardless of synthesis duration (figure 13). Like Bi_2_Se_3_ samples, Bi_2_Te_3_ remained as the main phase component. However, the maximum phase fraction achieved for the telluride was only 57.5 (4) wt% after 120 s heating with the tellurate phase steadily increasing over time.

There are several potential reasons for the continued prevalence of oxygen-containing impurities in these samples. Some delay between synthesis and analysis with post-synthesis handling in air could allow a (seemingly mildly) air-sensitive sample to oxidize. It is also possible that contact between the powdered starting materials and the interior surface of the silica ampoule could lead to a reaction with SiO_2_ during MW irradiation, as despite the low-relative permittivity of SiO_2_, the heat generated from the reactants would still be considerable. The observed deposition of what appears to be chalcogen or chalcogenides on the ampoule walls would result in an excess of Bi, thus, reducing the percentage of Bi_2_Se_3_ produced with extended heating. There is a further possibility that the oxygen partial pressure within the reaction ampoule was not negligible, either through insufficient evacuation or sealing. If in the Bi–Se system, Bi_2_SeO_2_ is formed through reaction with oxide at the tube walls then the quantity should be limited by the contact surface area between the reactant powders and the ampoule. Bi_2_Se_3_ will oxidize to Bi_2_SeO_2_ in the presence of oxygen when heated conventionally at 400℃, so under MW-heating conditions may be produced early before decomposing as the accelerated heating raises the temperature higher [44]. The production of BiSe over longer synthesis durations results in a marked reduction in the resulting Bi_2_Se_3_ phase fraction, suggesting that limiting the synthesis duration to less than or equal to 90 s would maximize Bi_2_Se_3_ production. In the Bi–Te system, it is interesting to note that heating results in the formation of a tellurate (Bi_2_TeO_5_) rather than an oxytelluride (Be_2_TeO_2_), which suggests a greater degree of oxidation occurs. The steady increase in Bi_2_TeO_5^-^_phase fraction with time could indicate that the oxide was progressively growing at the interface with the silica ampoule; with Bi progressively forming both the tellurate and telluride, remaining Te starting material is consumed relatively slowly over extended heating times. In air, Bi_2_TeO_5_ has been shown to oxidize to Bi_2_TeO_6_ at approximately 450℃ with further decomposition to TeO_2_ from 663℃, which itself breaks down above 790℃ [45]. The apparent absence of the tellurate (VI) in our system implies a restricted supply of oxygen. For the purposes of improving the phase purity of the target Bi_2_Te_3_ material, the use of an inner container material (e.g. inert reaction crucible) should eliminate the formation of oxides should the gettering of oxygen from SiO_2_ be responsible. However, for *in situ* experiments such an arrangement unfortunately presents further unwanted scattering.

### (d) *In situ* neutron diffraction studies of Bi_2_Se_3_ and Bi_2_Te_3_ syntheses

As with the antimony selenide *in situ* experiments, two 5 g samples of 2 : 3 Bi : X were prepared for each of X = Se and X = Te. The samples were heated for either *ca* 60 s or *ca* 120 s from the onset of accelerated heating. The heating times were recorded precisely in each experiment. For X = Se, each sample was successfully heated using 500 W forward power directly. However, despite achieving an improvement in coupling when using the DAQ and larger amounts of material, the induction period of the Bi_2_Te_3_ samples remained several minutes longer than the selenide. The Bi_2_Te_3_ samples were therefore heated using 100 W forward power with the aid of a graphite susceptor to reduce the induction period considerably (which even so was greater than 4 min).

### (e) Bi_2_X_3_ (X = Se, Te); *ca* 60 s heating regime

**Figure 14.**
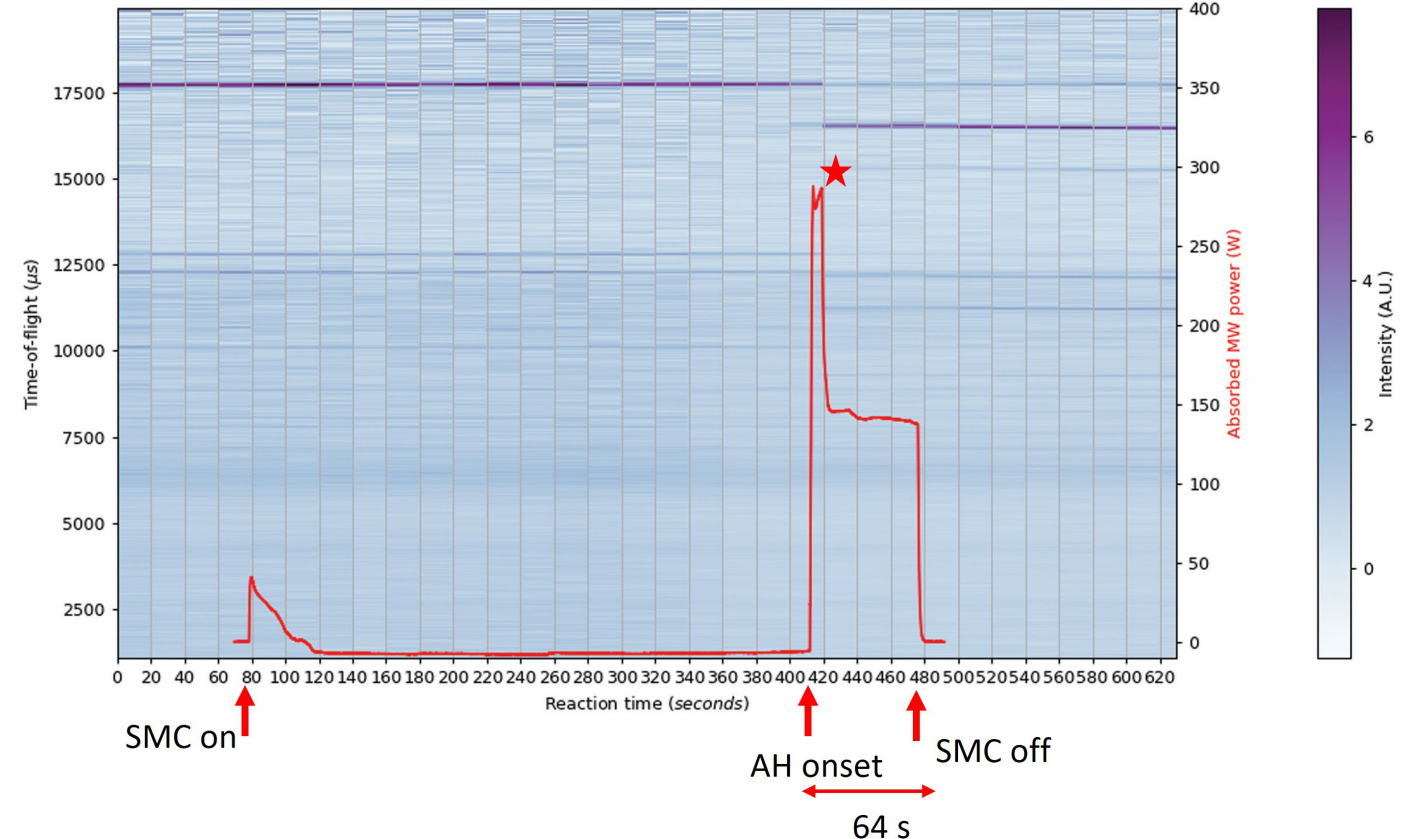
Contour colourmap plot of event mode TOF data (shades from blue to purple) on the left y-axis and absorbed MW power profile (red datapoints) on the right y-axis as a function of 20 s slices over the full experiment duration for the 64 s synthesis of Bi_2_Se_3_ using 500 W forward power—the red star indicates the onset of a new phase formation at 5−10 s. The intensity of the diffraction peaks is signified by the colour histogram on the right of each plot.

The power absorption profiles obtained using the DAQ give some valuable insight regarding the reaction pathways of the system when overlayed with the *in situ* neutron data. The Bi_2_Se_3_ sample irradiated at 500 W (figure 14), demonstrated rapid absorption of approximately 60% of the MW forward power corresponding to the onset of the accelerated heating period. The profile peaks at this value for only 5−10 s before falling to a consistent plateau at *ca* 150 W aligned with the formation of the selenide (and oxyselenide) product(s) almost instantaneously.

**Figure 15.**
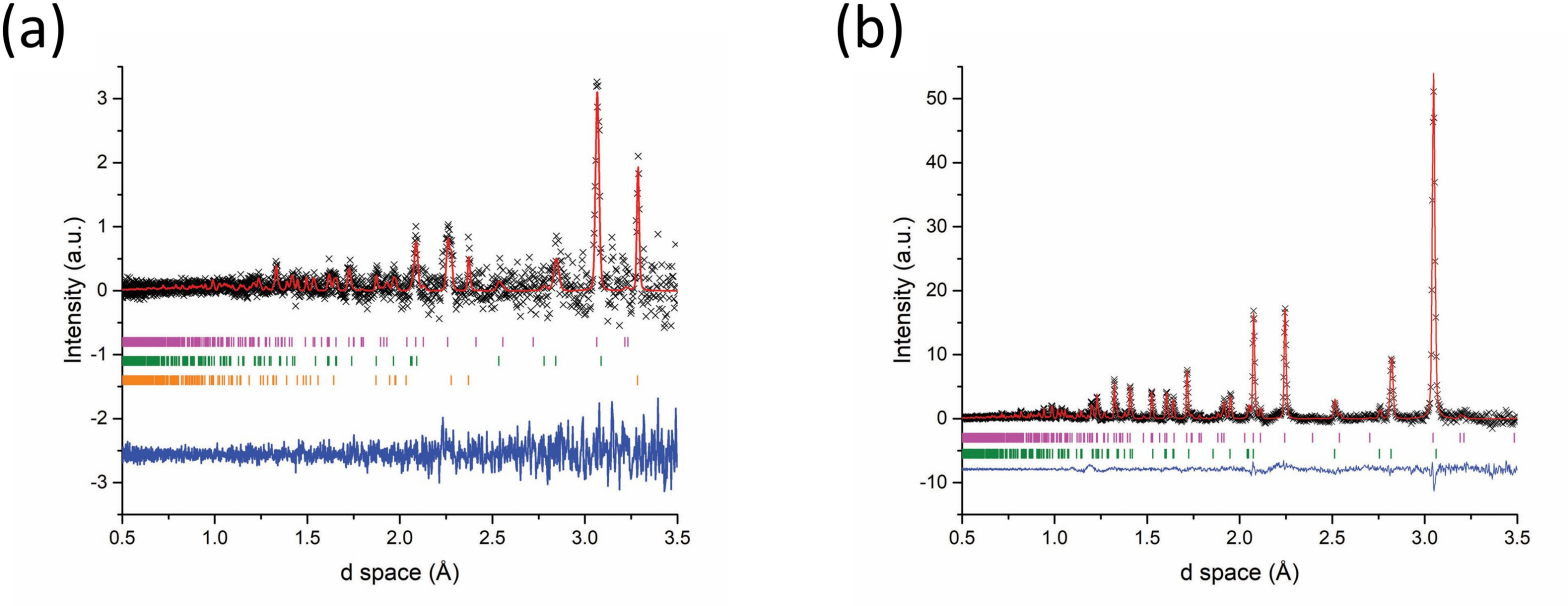
Rietveld fits to TOF data collected from Bi_2_Se_3_ synthesized using 500 W forward power for 64 s. Key: x -observed data, red plot - calculated fit, blue plot - difference profile: (a) Diffraction pattern generated from Event Mode data containing only those neutrons counted within the period of time 60−80 s after accelerated heating—Bragg reflection markers: magenta markers - Bi_2_Se_3_ (*R*$\bar{3}$*m*), green markers **-** Bi_2_SeO_2_ (*I4/mmm*) and orange markers - Bi (*R*$\bar{3}$*m*); (b) Histogram mode dataset collected for a period of 5 min after the reaction had completed. Bragg reflection markers: magenta markers - Bi_2_Se_3_ (*R*$\bar{3}$*m*) and green markers - Bi_2_SeO_2_ (*I4/mmm*).

Fitting the Event Mode data in the 60−80 s time slice period following accelerated heating showed three phases present: Bi_2_Se_3_ at 52.6 (1) wt%, Bi_2_SeO_2_ at 18 (3) wt% and unreacted Bi at 29 (3) wt% (figure 15a). Bi_2_SeO_2_ appears to have formed rapidly but continued to react with the Bi in the sample until the latter was fully expended and the final composition produced was a dual-phase system of Bi_2_Se_3_ (77.6 (2) wt%) and Bi_2_SeO_2_ (as shown in the refined post-reaction histogram data in figure 15b). The extreme step change in the MW absorption plot that marks the beginning and end of the reaction potentially presents an opportunity to automate the heating process (duration) based on real-time monitoring of MW power data.

**Figure 16.**
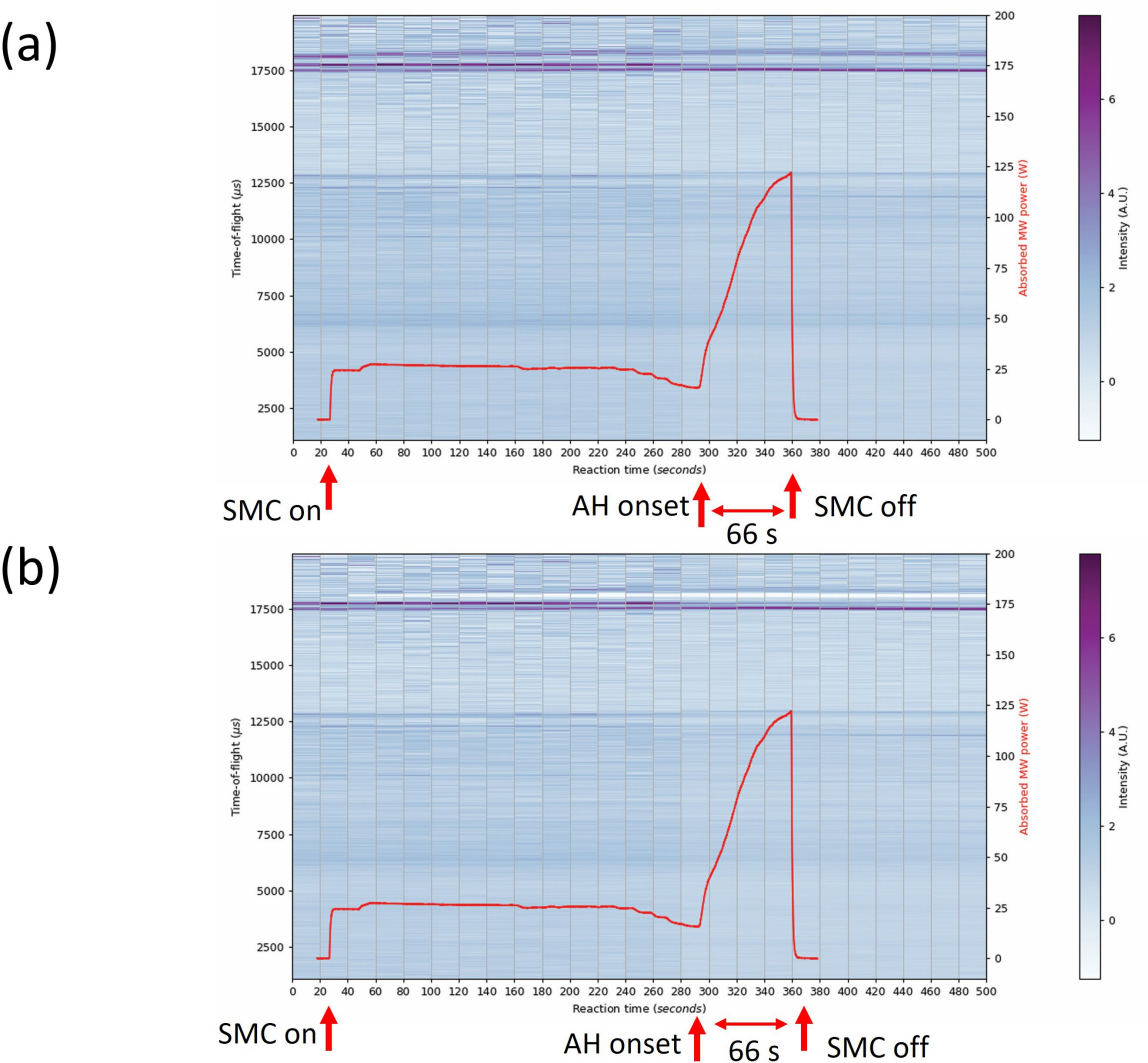
Contour colourmap plots of event mode TOF data (shades from blue to purple) on the left y-axis and absorbed MW power profile (red datapoints) on the right y-axis as a function of 20 s slices over the full experiment duration of 64 s synthesis of Bi_2_Te_3_ using 100 W with the aid of a graphite susceptor (a) data processed subtracting the background profile of the empty WG + silica tube; (b) data processed with the additional subtraction of a background profile from the graphite susceptor. The intensity of the diffraction peaks is signified by the colour histogram on the right of each plot.

The Bi–Te experiments were the only ones to be performed with a graphite susceptor in the SMC *in situ* reactor (figure 16). For the purposes of data analysis, a background histogram file of an empty silica ampoule containing an approximately 5 mm depth of graphite powder in the silica support tube was recorded before the experiments and subtracted during data normalization. Although largely successful, this approach presented a region of anomalously low scattering when subtracted from the recorded data (figure 16b) in the ToF range of 18 000−18 2000 µs. This is somewhat inevitable from this approach given that differing amounts of graphite susceptor are likely to be present in the neutron beam during different measurements. Furthermore, as the temperature increases the position of graphite peaks will shift to higher d-spacing (lower Q) as the crystal structure expands, particularly along the *c*-axis [46,47]. This means that measurements made on room temperature graphite for background subtraction will not necessarily successfully remove the respective peaks for *in situ* data collected at variable temperatures. The absorbed power profile for the sample heated with the aid of a susceptor contrasts markedly with profiles from direct MW heating. Rather than a step change marking the onset of accelerated heating, the characteristic plot shows a steady increase in lossy behaviour as the local temperature increases. Graphite has an extremely high melting point of 3600℃ meaning that although the *ε*″ parameter can increase exponentially with temperature, the material remains solid and crystalline throughout [48]. At approximately 40 s after the start of accelerated heating, the rate of increase of the absorbed power drops dramatically as it approaches the maximum absorbance. Although only 100 W forward power was used, the error associated with the measurement of the reflected power tended to be high, particularly as the dielectric properties of the load fluctuated. As a result, the calculated absorbed MW power appears to reach a limit of 125 W, and the value would probably increase further on approaching the plateau of maximum absorbance if heating were allowed to continue. The peak at approximately 17 700 µs can be clearly seen to fade in intensity just before accelerated heating (280–300 s section of figure 16) and to continue to lose intensity throughout the heating cycle and following cooling period. This indicates a decrease of Te in the sample as it reacts and is consumed, leaving only Bi_2_Te_3_ and unreacted Bi in the final mixture.

**Figure 17.**
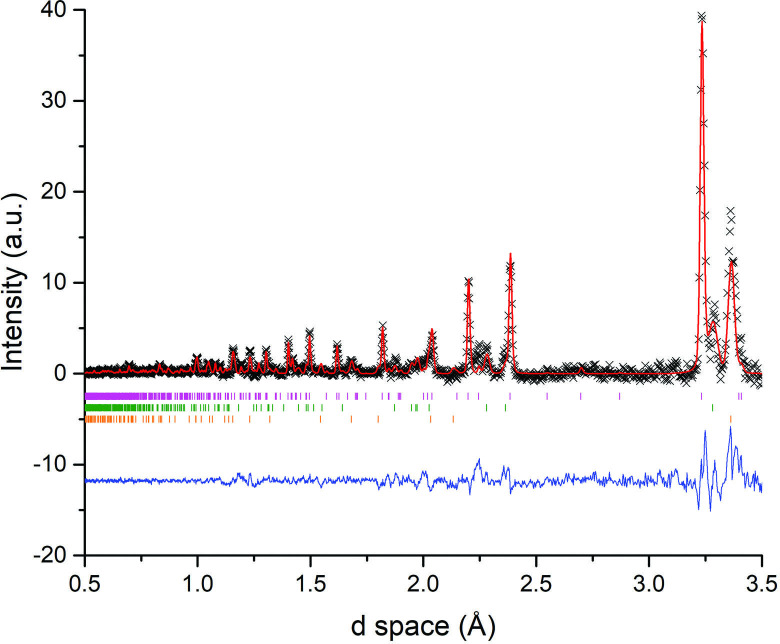
Rietveld fit to a diffraction pattern generated from Event Mode ToF data collected during Bi_2_Te_3_ synthesis using 100 W forward power for 64 s and using a graphite susceptor. The dataset contains only those neutrons counted within the 0−20 s time interval following the onset of accelerated heating. Key: x - observed data, red plot - calculated fit, blue plot - difference profile—Bragg reflection markers: magenta markers - Bi_2_Te_3_ (*R*$\bar{3}$*m*), green markers - Bi (*R*$\bar{3}$*m*) and orange markers - C (*P6_3_mmc*).

Despite the removal of some sample diffraction peaks that overlap with graphite reflections as a result of the subtraction process, the remainder (vast majority) of the pattern could be indexed and/or fit by Rietveld refinement. Analysis of the data was concentrated on the first 20 s after the onset of accelerated heating. The data reveal that reaction initiation appears to be almost instantaneous from this onset point, with Bi_2_Te_3_ identified in the first 5 s accompanied by the near-disappearance of Te. The phase fraction of Bi_2_Te_3_ in the period of 0−20 s after accelerated heating was determined from Rietveld structure refinement to be 65 (2) wt%, with unreacted Bi forming the balance of the sample. The fraction of Bi_2_Te_3_ improved to 91.5 (2) wt% according to the post-reaction histogram data (figure 17); Te is consumed with the remainder probably deposited on the ampoule walls, leaving an amount of unreacted Bi in the solid product.

### (f) Bi_2_X_3_ (X = Se, Te); *ca* 120 s heating regime

**Figure 18.**
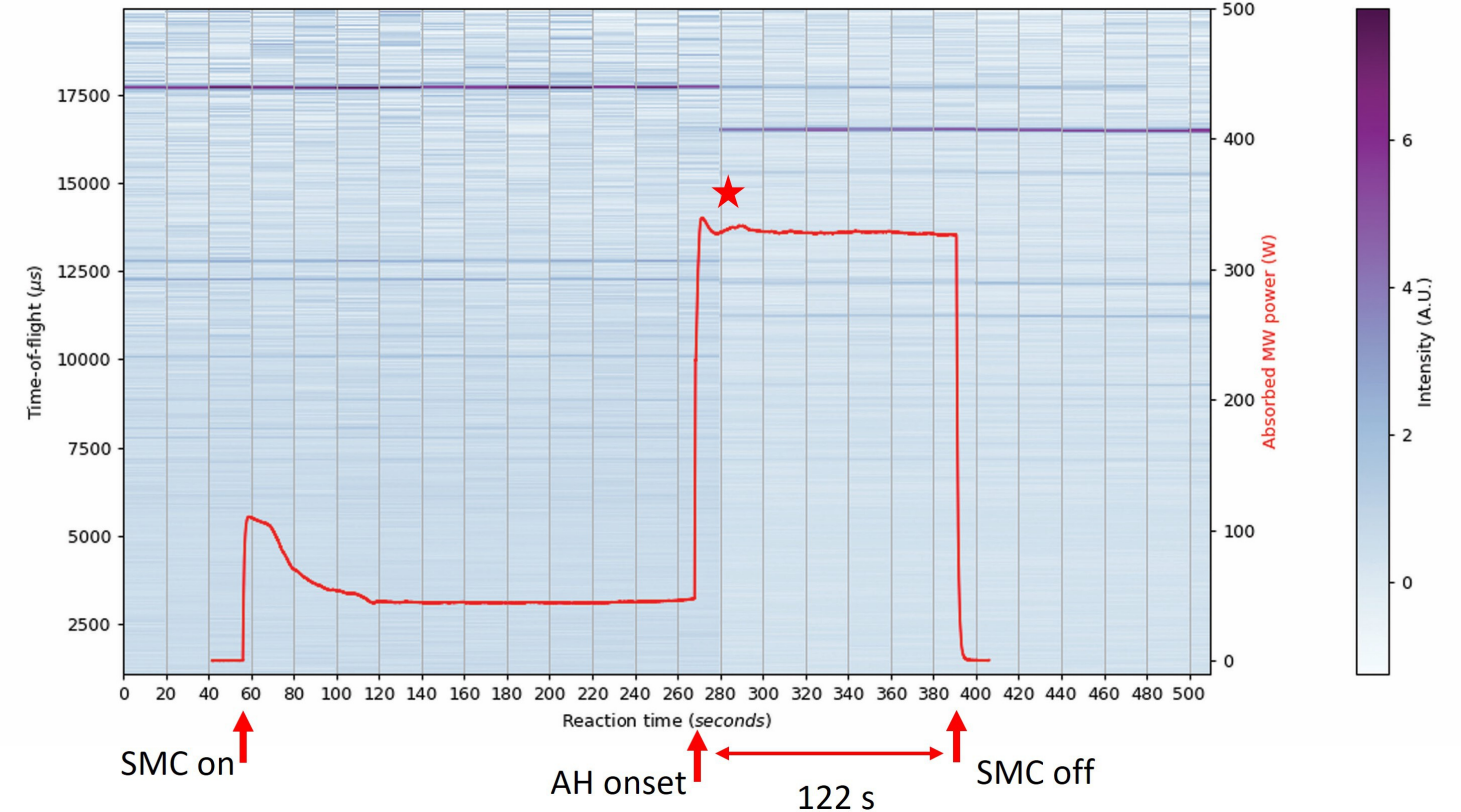
Contour colourmap plot of event mode TOF data (shades from blue to purple) on the left y-axis and absorbed MW power profile (red datapoints) on the right y-axis as a function of 20 s slices over the full experiment duration of 122 s synthesis of Bi_2_Se_3_ using 500 W. The intensity of the diffraction peaks is signified by the colour histogram on the right of each plot.

Although benchtop experiments indicated that limiting the heating cycle to less than or equal to 90 s following the onset of accelerated heating should maximize Bi_2_Se_3_ production, it was considered that by following the process beyond 90 s we would not only be able to confirm the formation of the desired phase but also to determine any subsequent events (such as decomposition) on extended heating. In addition, benchtop experiments for X = Te (using a graphite susceptor) had suggested that production of the chalcogenide had not yet peaked at 90 s. As noted with the X = Se sample heated for approximately 60 s, rapid synthesis for the 120 s sample was observed to occur within 5−10 s of the onset of accelerated heating. This is clear at 280 s in figure 18, where the distinctive peak at approximately 17 500 µs abruptly reduces in intensity close to the onset of accelerated heating and disappearing completely approximately 90 s after MW power was switched off while the sample cools. The absorbed power reaches a maximum stable plateau at 340 W until the MW power is switched off after 122 s. Over the same time period a strong peak at approximately 16 200 µs indicates the rapid formation of Bi_2_Se_3_. The peak continues to grow in intensity as the sample cools and presumably crystallizes.

**Figure 19.**
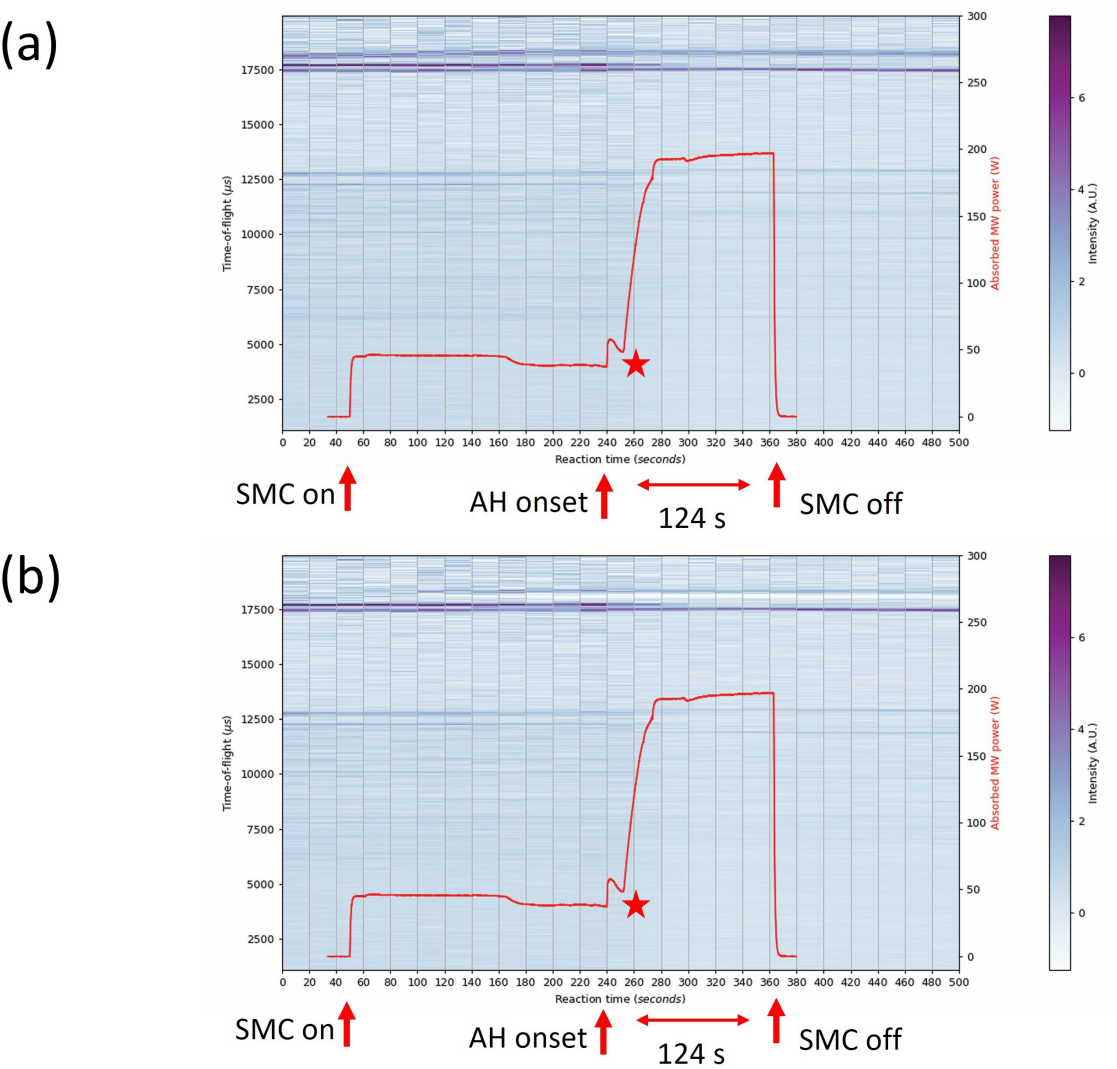
Contour colourmap plots of event mode TOF data (shades from blue to purple) on the left y-axis and absorbed MW power profile (red datapoints) on the right y-axis as a function of 20 s slices over the full experiment duration of 122 s synthesis of Bi_2_Te_3_ using 100 W with the aid of a graphite susceptor (a) data processed subtracting background profile of empty WG + silica tube (b) data processed subtracting background profile of graphite sample. The red star indicates the event 15 s into accelerated heating where the graphite susceptor appears to become the principal absorber. The intensity of the diffraction peaks is signified by the colour histogram on the right of each plot.

The absorbed MW power profile for the Bi_2_Te_3_ 124 s synthesis (figure 19) follows a similar path to the sample heated for 64 s (with both using a graphite susceptor), although the absorption rate of the susceptor from the accelerated heating onset is somewhat higher. In the 124 s synthesis, the power absorption reaches the maximum (plateau) value of approximately 40 s from the onset of accelerated heating. Although the profile appears to reach an absorbed power of 200 W, only 100 W of MW power was used to heat the sample, and so the calculated value of the absorbed power cannot be accurate. Nevertheless, the shape of the plot indicates virtually complete absorption of MWs by the sample load. The smaller peak in power absorption at the early stage of accelerated heating (peaking at 50 W at approx. 240 s in figure 19) could relate to the start of Bi_2_Te_3_ synthesis (or at least coupling of the field with the Bi and Te reactants) rather than representing an artefact relating to the graphite heating, since the profile otherwise tends to subsequently follow a curve more closely matching that of the 64 s synthesis (suggestive of the contribution of the susceptor).

**Figure 20.**
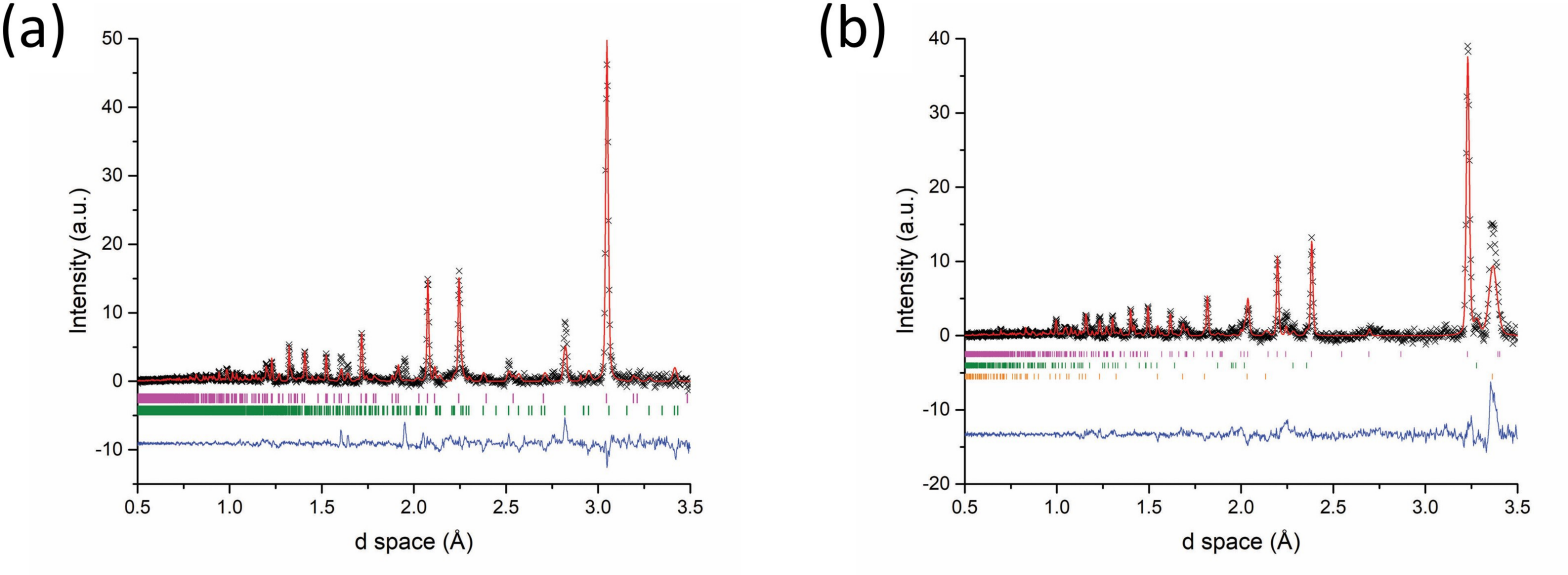
Rietveld fits to Histogram mode ToF data collected for 5 min immediately after the specified reaction had completed. Key: **x** - observed data, red plot - calculated fit, blue markers - difference profile: (a) Bi_2_Se_3_ synthesized using 500 W forward power for 122 s—Bragg reflection markers: magenta markers - Bi_2_Se_3_ (*R*$\bar{3}$*m*), green markers - Bi_2_Se_3_ (*Pnma*); (b) Bi_2_Te_3_ synthesized using 100 W forward power, employing a graphite susceptor for 124 s—Bragg reflection markers: magenta markers - Bi_2_Te_3_ (*R*$\bar{3}$*m*), green markers - Bi (*R*$\bar{3}$*m*) and orange markers - C (*P6_3_mmc*).

The refined phase fractions of the post-reaction histogram data (figure 20) confirmed that extending the heating duration did not improve upon the phase purity of either sample. In the first 20 s after the onset of accelerated heating Bi_2_Se_3_ was identified at 39 (4) wt% while in the next 20 s Bi_2_SeO_2_ appeared although phase fractions could not be refined. The post-reaction histogram (figure 20a) indicates a product comprised of two polytypes of bismuth selenide; 63.5 (5) wt% of rhombohedral (space group *R*$\bar{3}$*m*) Bi_2_Se_3_ and 36.5 (5) wt% orthorhombic (*Pnma*) Bi_2_Se_3_. Interestingly, the oxychalcogenide phase was only identified during the heating phase and appears to have reacted over the longer heating cycle yielding only Bi_2_Se_3_ (figure 20b). The implication is that extended heating can yield products free of the Bi_2_SeO_2_ phase, probably owing to the higher temperature reached. The extended heating regime may also be responsible for the appearance of the orthorhombic phase of Bi_2_Se_3_, which is metastable and typically forms on relaxing from high to ambient pressure [49]. It is not obvious from the Event Mode data at which time the orthorhombic phase first appears (and whether it is linked to the consumption of the Bi_2_SeO_2_ phase) and this would be another topic worthy of further study.

For each of the Bi_2_X_3_ (X = Se, Te) systems, the *in situ* neutron studies show that direct MW heating can produce the desired compounds, but not at 100% phase purity. For X = Te, it is evident that the synthesis remains incomplete within the applied heating and cooling cycle and that a direct (i.e. susceptor-less) route would still be preferred for which reaction conditions could be further optimized. The X = Se syntheses are more successful and phase purity is prevented only by the existence of Bi_2_Se_3_ polymorphs. This in itself is an intriguing finding that could be developed positively if conditions for phase-selective synthesis can be determined.

## 4. Conclusions

Studies on several gram-scale syntheses of Sb_2_Se_3_, Sb_2_Te_3_, Bi_2_Se_3_ and Bi_2_Te_3_ have demonstrated the utility of a new SMC MW reactor constructed specifically to perform *in situ* solid-state reactions on a neutron beamline. Reactions initially trialled using a bespoke SMC benchtop reactor were transferred successfully to a configuration ideally suited to time-resolved powder neutron diffraction on the instrument Polaris. A combination of developments in both hardware and software has made these experiments possible. By carefully recording the MW power in the WG using a DAQ module, time-dependent events such as chemical reactions and phase transitions can be inferred from the MW absorption data. Coupling these absorption data with time-resolved diffraction data over choreographed timeslices using revolutionary Event Mode processing provides a powerful technique that allows solid-state MW reactions to be probed *in situ* in real time for the first time. In this way, rapid transitions from reactants to products can be correlated with the step changes in MW field coupling that signify accelerated heating. It is important to stress that with Event Mode data collection it is only possible to generate useful diffraction patterns (i.e. of sufficient statistical quality and with time slice increments fine enough to follow the reaction pathway) from which to extract meaningful phase and structural information if the scattering from the samples and intensity of the neutron source produces a high enough count rate in the detectors.

The reactor commissioning experiments provided a large number of datasets within a very short period of time. The preliminary investigations demonstrated that the nature of the chemical reaction pathways (for example, changes of state, potential evolution of gases) had a strong influence on the quality of the data obtained. Despite these limitations, the set-up was able to adapt to the rapidity of the MW-induced reactions. Although the low signal : noise ratio in many of the powder patterns extracted from Event Mode datasets currently prevents comprehensive structure refinement, phase identification is achievable and better than semi-quantitative phase composition data are obtainable. Although operating with a small sample size at present, already *in situ* experiments have demonstrated the advantages of the technique over *ex situ* diffraction methods. Chalcogenide products evidently form through crystallization during sample cooling and combined contour colourmaps provide the first reliable evidence of reaction pathways from periods of accelerated heating through to sample cooling. In this respect, the Sb-X and Bi-X systems are notably different; the former requires extended heating during which the sample progresses through two plateaus of absorbed power while the latter reacts almost instantly and fully forms (crystallizes) over extended cooling.

Although the presence of oxygen-containing phases (such as oxychalcogenides, M_2_XO_2_ and bismuth tellurate, Bi_2_TeO_5_) in samples was not planned, their presence revealed unexpected information regarding their decomposition/reaction steps to other intermediate and product phases. Indeed, with the benefit of benchtop experiments preceding *in situ* studies, synthesis times could be optimized to drive the formation of intermediate Bi_2_SeO_2_ through to Bi_2_Se_3_ at completion by extending heating beyond 60 s. Also in the Bi–Se system, *in situ* experiments revealed that the extreme conditions yield the metastable orthorhombic form of Bi_2_Se_3_ which normally only persists under high-pressure conditions.

The *in situ* SMC reactor is thus a powerful tool for interrogating the rapid (and sometimes volatile) nature of MW-induced reactions between solid reactants. Further improvements will doubtless enable the value of extracted data to continue to grow; these could relate equally to the reactor and to data handling capabilities. Reducing incident MW frequency (to 915 MHz, for example) could slow heating/reaction rates for the benefit of diffraction signal : noise ratio and counting statistics while also maximizing MW skin depth. Ultimately, *in situ* experimental MW synthesis profiles could be collated into a database of materials system performance information for both academic and commercial use. Moreover, based on adjustments to reactant stoichiometry, incident MW power and synthesis duration it should be possible to not only produce certain materials to order but also to tune the structures and therefore the properties and performance of particular materials selectively.

## Data Availability

Data supporting this article have been included as part of the electronic supplementary material. Data files may also be obtained from https://doi.org/10.5286/ISIS.E.RB2030041 [50]. Supplementary material is available online [51].
